# Rare CNVs in Suicide Attempt include Schizophrenia-Associated Loci and Neurodevelopmental Genes: A Pilot Genome-Wide and Family-Based Study

**DOI:** 10.1371/journal.pone.0168531

**Published:** 2016-12-28

**Authors:** Marcus Sokolowski, Jerzy Wasserman, Danuta Wasserman

**Affiliations:** 1 National Centre for Suicide Research and Prevention of Mental Ill-Health (NASP), Karolinska Institute (KI), Stockholm, Sweden; 2 WHO collaborating Centre for research, methods, development and training in suicide prevention, NASP, KI, Stockholm, Sweden; Universitat Wien, AUSTRIA

## Abstract

Suicidal behavior (SB) has a complex etiology involving genes and environment. One of the genetic components in SB could be copy number variations (CNVs), as CNVs are implicated in neurodevelopmental disorders. However, a recently published genome-wide and case-control study did not observe any significant role of CNVs in SB. Here we complemented these initial observations by instead using a family-based trio-sample that is robust to control biases, having severe suicide attempt (SA) in offspring as main outcome (*n* = 660 trios). We first tested for CNV associations on the genome-wide Illumina 1M SNP-array by using FBAT-CNV methodology, which allows for evaluating CNVs without reliance on CNV calling algorithms, analogous to a common SNP-based GWAS. We observed association of certain T-cell receptor markers, but this likely reflected inter-individual variation in somatic rearrangements rather than association with SA outcome. Next, we used the PennCNV software to call 385 putative rare (<1%) and large (>100 kb) CNVs, observed in *n* = 225 SA offspring. Nine SA offspring had rare CNV calls in a set of previously schizophrenia-associated loci, indicating the importance of such CNVs in certain SA subjects. Several additional, very large (>1MB) sized CNV calls in 15 other SA offspring also spanned pathogenic regions or other neural genes of interest. Overall, 45 SA had CNVs enriched for 65 medically relevant genes previously shown to be affected by CNVs, which were characterized by a neurodevelopmental biology. A neurodevelopmental implication was partly congruent with our previous SNP-based GWAS, but follow-up analysis here indicated that carriers of rare CNVs had a decreased burden of common SNP risk-alleles compared to non-carriers. In conclusion, while CNVs did not show genome-wide association by the FBAT-CNV methodology, our preliminary observations indicate rare pathogenic CNVs affecting neurodevelopmental functions in a subset of SA, who were distinct from SA having increased SNP risk-allele burden. These observations may open up new avenues in the genetic etiology of SB.

## Introduction

Suicidality involves a substantial genetic component, as shown by family, adoption and twin studies; heritability is in the ranges between 30–55% [1, 2]. The etiology is partly overlapping among suicidal behaviors (SB) of different severities, i.e. suicide attempts (SA) or completed suicides. Psychiatric disorders are observed in 90% of suicides, e.g. schizophrenia, bipolar disorder and major depression, but sufficient knowledge about specific factors is lacking [3]. SB etiology is also studied through various endophenotypes relating to behavior-/stress-dysregulation and neurocognitive changes [3, 4]. A neurodevelopmental origin of SB appears to be generally supported [5]. To study the genetics of SB, candidate genes have often been investigated in a monogenetic manner. But it is increasingly clear that the overall genetic etiology of SB is most likely polygenic, thus involving many genes and genetic variants of small effects across several neurosystems [6–10].

The few reported SNP-associations reported from case-control genome-wide association studies (GWAS) on SB [10–14] are yet quite inconclusive [9]. One might instead hypothesize that copy number variation (CNV) is of greater importance in SB and easier to detect. However, a recent genome-wide and case-control study of CNVs in SB failed to show any significant findings [15], perhaps indicating a lesser role for CNVs than has been observed for e.g. neurodevelopmental disorders [16]. We thought it would be of interest to complement on those initial observations [15] by using a family-based trio-sample that is robust to control biases, having severe suicide attempt (SA) as main outcome in the offspring (*n* = 660 trios) [17–26]. This sample has been used previously by us in both candidate gene [17–26] and also GWAS [27] investigations. We first tested for common CNV associations by FBAT-CNV, without relying on any CNV calling algorithm. We then called rare CNVs in SA offspring by using PennCNV and investigated features of potential interest which has not been previously described for SB. For example, while polygenic SNP risk-alleles have been reported to overlap between SA and schizophrenia [10, 27], the overlap of specific rare and large CNVs is yet unknown [15], whereby we here described the occurrence of well-studied and previously schizophrenia-associated CNVs in SA. Other CNVs of putative interest and their enriched biological functionality were also characterized, that is >1 MB sized CNVs which may have increased pathological potential, as well as all CNVs that intersected with medically relevant genes [28]. Finally, we also investigated if rare and large CNVs coexisted with increased load of SNP risk-alleles [27] in the same SA subjects. This report was guided by the STREGA recommendations [29].

## Materials and Methods

### Research subjects and the suicide attempt (SA) main outcome

Research subjects and SA main outcome have been extensively described in our previous candidate gene association studies, as part of the Genetic Investigation of Suicide Attempt and Suicide (GISS) project [17–26]. Briefly, nuclear family trios (all complete with both biological parents and one SA offspring per trio; n = 660), were collected in Ukraine by recruiting the offspring from emergency care due to an SA as defined by a score of ≥ 2 on a Medical Damage Rating Scale (MDS) [30], which represented the primary ascertainment criteria for inclusion. An MDS of ≥ 2 ensured a minimum level of self-inflicted injury, e.g flesh wounds, first degree burns or moderate bleeding; 45% of our SA had MDS ≥ 4 which represents a significant danger to life sufficient for inpatient hospitalization. Other aspects of the sample recruitment, selection criteria, demographics, ancestry and International Classification of Diseases 10th ed. (ICD-10) psychiatric diagnoses have been described in detail previously [17–20] and the predominant diagnoses are briefly outlined in Table 1. The collection of research subjects followed the code of ethics of the World Medical Association (Declaration of Helsinki), and written consent was obtained. The study was approved by the Research Ethics Committee at the Karolinska Institute (Dnr 97–188) and by the Ministry of Health in Ukraine.

**Table 1:** Sample description.

	SA offspring	Parents
Age	24.2 ± 7.2	51.1 ± 8.6
Male	337	660
Russian / Ukrainian ethnicity	97.6%	99.5%
Medication	139	n/a
Schizophrenia / BPD	88	7
Major depression	85	18
Posttraumatic stress disorder (PTSD)	109	34
Suicide attempt (SA)	660	36
Total	660	1320

Age is shown as mean ± standard deviation. Plain numbers refers to the amount of subjects. Ethnicity refers to the origin of the parents (or parent’s parents). Medication refers to any psychotropic medication used prior to the index SA, mainly antidepressant or antipsychotic drugs. Diagnoses were according to the International Classification of Diseases 10th ed. (ICD-10). “Schizophrenia / BPD” were schizophrenic (“SCZ”; ICD-10 code F20, n = 59), schizoaffective (F25, *n* = 4), delusional (F22, *n* = 7), psychotic (F23, *n* = 18), bipolar disorder (“BPD”; F31, *n* = 6) and/or depression with psychotic symptoms (F33.3, *n* = 4). Major depression SA had moderate or severe depression diagnoses (F32-33).The most prevalent anxiety-type diagnosis in the sample was posttraumatic stress disorder (“PTSD”; F43.1), which was comorbid with the other diagnoses as follows: 8% in SCZ and 14% in MDD. Only phenotype(s) of SA offspring are invoked in the family-based CNV statistic.

### DNA preparation, genotyping and quality control filtering

Venous blood (10 ml) was taken from all research subjects into EDTA-containing tubes. DNA isolation was performed as described previously [31]. SNP genotyping was done using the HumanOmni1-Quad_v1 chip (Illumina Inc.) at the SNP&SEQ Technology Platform facility (www.molmed.medsci.uu.se), assaying ~1 million SNPs with each trio plated consecutively. For the raw data, 96.7% of SNPs had a call rate >99%, >99.99% of calls were reproducible, >99.99% of family-wize calls had no mendelian errors, and duplicated individuals could be ruled out. SNPs were QC-filtered to obtain call rates > = 95%, hardy weinberg equilibrium (HWE) exakt *P* ≥ 10^−6^, minor allele frequency (*MAF*) ≥ 0.01 and no mendelian errors, whereby 739,780 autosomal and 17,501 X-chromosomal SNPs remained. Autosomal SNPs were LD pruned with *r*^*2*^-threshold 0.8, which yielded 449,256 SNPs available for analyses. Quantiles vs quantiles (Q-Q) plots of SNP-allelic transmission *P*-values showed that the observed followed the expected uniform null (genomic inflation factor λ = 1.002), as we have recently shown elsewhere [27].

### Copy number variant (CNV-) based GWAS

We tested for autosomal associations of putative CNVs directly (without any prior CNV calling algorithm or CNV QC), using the raw intensity (Illumina “log R ratio”, LRR) values of either 88,450 CNV markers or the 449,256 post-QC SNPs, by an CNV-adapted family-based association test (FBAT) [32] with default settings, as implemented in the PBAT analysis package in SVS 8.0 (Golden Helix, Bozeman, Montana, USA). The CNV markers are used to detect known CNVs, while the SNPs might additionally detect unknown CNVs (or be used to support the observed CNV marker associations).

#### Multiple testing

For the 88,450 on-chip CNV-loci of the HumanOmni1-Quad_v1 chip, we used an experiment-wide, Bonferroni-corrected significance threshold of *P* ≤ 5.6 x 10^−7^. *P* < 5 x 10^−8^ was the genome-wide significant threshold [33]. Only nominal (uncorrected) *p*-values are reported.

#### Power

The power of the FBAT-CNV method is similar to ordinary TDT for SNPs [34]. Statistical power was calculated by using software QUANTO v.1.2.5 (http://hydra.usc.edu/gxe/) [35]. The study had 80% power (assuming α = 5.6 x 10^−7^, log-additive model, n = 660 trios, population risk = 0.01) to detect CNVs with effects down to OR≥1.6 (if MAF = 0.49), but required OR≥2.0 for MAF = 0.1.

### Rare and large CNVs

We focused on putative rare (<1%) and large (>100 kb) autosomal CNVs in SA, i.e. the CNVs which are more reliably detected as well as implicated in developmental pathologies [16]. The LRR of all QC-passed SNPs and 88,450 CNV probes with <5 missing values were used. Using PennCNV [36], we called CNVs for each individual on GC wave corrected LRR data, and then used the output for trio-based calling, keeping only CNVs called with ≥ 30 markers and ≥ 100 kb size for high confidence. QC with *filter_cnv*.*pl* resulted in removing 43 trios due to ≥ 50 CNV calls or *LRR_SD* > 0.3 in ≥1 of the family-members, as well as removing 35 trios which had ≥ 10 rare CNV calls in ≥1 of the family-members (Table B in S1 File). Calls located in centromeric, telomeric, immunoglobulin or T-cell receptor regions were then also removed as recommended (Table B in S1 File). To obtain rare CNVs, we used BEDTools [37] to group CNV calls that overlapped or located within 20kb of each other into unique IDs, and then kept the CNVs which were observed with frequency ≤ 1%. We finally removed CNV calls which overlapped by >50% with segmental duplications and merged adjacent CNV calls into larger CNVs (if gaps were <20% of CNV length). Among the final 385 putative CNVs in *n* = 225 remaining SA CNV carriers (47% males), 49%/51% were deletions/duplications, respectively (Tables B and C in S1 File). 146 SA carried 1, 51 SA carried 2, 13 SA carried 3, 3 SA carried 4, 4 SA carried 5, 2 SA carried 6, 4 SA carried 7, 1 SA carried 8 and 2 SA carried 9 putative CNVs, respectively.

Among SA CNV carriers, we evaluated the burden of CNVs in previously schizophrenia-associated loci [38], ≥1MB sized CNVs as well as CNVs intersecting with a list of medically relevant genes shown to involve CNVs [28]. For gene to CNV mapping we used a set of 19897 Ensembl and Entrez consensus genes, as before [27]. We did not have controls for association testing (e.g. unaffected sibs), but CNV burden was compared to previously published control samples in 2x2 tables, using odds ratios and Fisher’s exact p-values (two-tailed). If there was zero counts in a cell, a standard continuity correction was applied by adding 0.5 to each cell of the 2x2 table [39], as before [40]. CNV burden frequencies were calculated in relation to the *n* = 582 SA in our sample, whom had passed sample QC for CNVs. For combined analysis of several previously published samples (e.g. Table 2), we performed meta-analysis on the odds ratios by help of WinPepi v.3.83 [41]. Gene set enrichment was tested by hypergeometric test or by submitting gene symbols to ToppGene [42], testing set sizes 20–2000 with Bonferroni correction. Protein-protein interaction (PPI) networks were examined on the STRING v.10 server [43].

**Table 2:** Burden of putative rare and large (>100 Kb) CNVs in schizophrenia-associated loci, with comparison to four previously published control sets.

Loci and CNV type	Chr#: start–stop (Mb)	#CNVs in SA (#*de novo*)	SA sex	#CNVs in control samples of previous studies (S)				SA *vs* controls: *P*-value, OR(95%CI)
				S ^1^	S ^2^	S ^3^	S ^4^	
1q21.1 del/dup	Chr1:145–145.9	0	*-*	1	6	1	0	0.20, 2.4(0.24–24)
NRXN1 del	Chr2:50–51.1	1 (1)	*m* ^M^	*n/a*	0	0	0	0.002, 70(5.7–849)
3q29 del	Chr3:197.2–198.8	0	*-*	0	0	0	0	0.024, 41(2.4–707)
WBS dup	Chr7:72.4–73.8	1 (1)	*f* ^S^	*n/a*	1	0	*n/a*	0.09, 21(2.2–202)
VIPR2 dup	Chr7:158.5–158.6	0	*-*	*n/a*	6	2	*n/a*	0.34, 1.2(0.12–12)
15q11.2 del	Chr15:20.4–20.6	1 (0)	*f* ^S^	40	26	0	5	0.99, 0.58(0.10–3.3)
AS/PWS dup	Chr15:22.4–26.1	0	*-*	*n/a*	0	*n/a*	0	0.07, 13(0.75–226)
15q13.3 del	Chr15:28.9–30.3	0	*-*	4	2	2	0	0.20, 2.4(0.24–24)
16p13.11 dup	Chr16:15.4–16.2	2 (0)	*m*,*m* ^P^	25	12	6	2	0.31, 1.8(0.49–6.8)
16p11.2distdel	Chr16:28.7–29	0	*-*	*n/a*	2	1	*n/a*	0.17, 3.0(0.28–32)
16p11.2 dup	Chr16:29.5–30.1	1 (1)	*m*^A^	4	0	2	0	0.16, 6.8(1.08–43)
17p12 del	Chr17:14.1–15.4	0	*-*	*n/a*	3	*n/a*	0	0.26, 1.9(0.17–20)
17q12 del	Chr17:31.9–33.3	1 (1)	*m* ^A^	2	0	0	0	0.07, 21(2.7–156)
22q11.2 del	Chr22:17.4–19.8	2 (2)	*f* ^A^,*f* ^M^	0	0	0	0	<0.001, 205(18–2281)
	Total:	9 (6)	-	76	58	14	7	
	Freq (%):	1.55	-	0.74	0.92	0.24	0.54	

Positions are in the NCBI36/hg18 assembly. See Fig C in S1 File for plots of genomic locations and LRR / BAF profiles. Del, deletion; Dup, duplication; SA, suicide attempter; *n/a*, data was not available; *m*, male; *f*, female.

^1^ Grozeva *et* al. [47], *n* = 10259 WTCCC controls.

^2^ Rees *et* al. [38], *n* = 6316 controls.

^3^ Szatkiewicz *et* al. [40], *n* = 5917 controls.

^4^ Chapman *et* al. [46], *n* = 1290 controls.

^S^ SA diagnosed with schizophrenia

^M^ SA diagnosed with MDD

^P^ SA diagnosed with PTSD

^A^ SA used psychotropic medications prior to the index SA

### Polygenic risk scores

**Fig 1:**
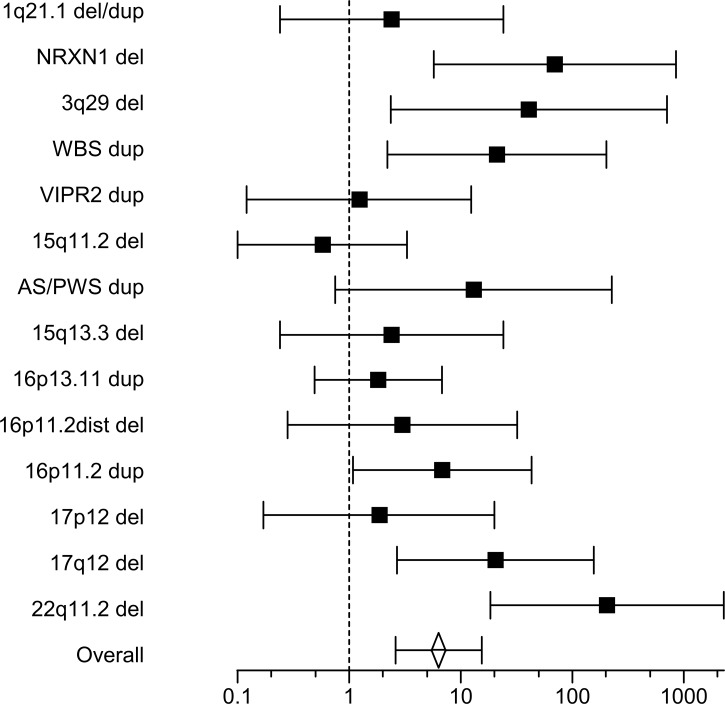
CNV burden of schizophrenia-associated loci in SA. Further details about the loci are listed in Table 2. Black squares depict the odds ratio (with 95% confidence intervals) for SA as compared to four merged control samples at each loci (Table 2), while the open diamond depict the overall random effects odds ratio across all 14 loci (see results text).

We followed up on our recently published results concerning the increased common SNP risk-allele burden (polygenic risk scores) in neurodevelopmental genes of SA (Fig 1A and 1B in ref [27]), by repeating the previous analysis in SA stratified into the “genetic subgroups” of *n* = 225 CNV carriers and *n* = 357 CNV non-carriers. The discovery and target samples were originally at *n* = 330 each [27]. Here we used the same risk-alleles in neurodevelopmental genes as originally defined by the discovery sample of *n* = 330 [27], but the target samples now consisted of either SA CNV carriers (*n* = 116) or CNV non-carriers (*n* = 172). To control for sample size differences, we also re-analyzed ten random draws of *n* = 116 from the CNV non-carriers and compared with the results for CNV carriers, using significance-testing by Student's t-test (two-tailed; provided normality by Shapiro–Wilk test *P*>0.05). The proportion of variation explained in SA by the polygenic risk scores (and the association), was evaluated by conditional logistic regression (CLR) and the Nagelkerke’s pseudo-*R*^2^ measure (using Stata v.9.2), at *P*_T_ < 0.1 as before [27].

## Results

We tested genome-wide association of CNVs with the SA outcome, similar to a SNP-based GWAS. The FBAT-CNV methodology detects association of any CNV type, without the need for any prior CNV-calling [32], and with similar power characteristics as ordinary SNP-based FBAT [34]. Among the 88,450 on-chip CNV-markers tested, two proximal markers at chromosome 14 (Illumina markers cnvi0108946 and cnvi0118308; Table A in S1 File) were experiment-wide significant (*P* ≤ 5.6 x 10^−7^; S1 Table). Using SNPs to complement the testing of CNV-markers, five genome-wide significant CNV-signals (*P* ≤ 5 x 10^−8^; S2 Table) were also observed (Table A in S1 File); four at chromosome 14 (rs8010032, rs10143357, rs17116313 and rs8016619) in the proximity of the significant CNV markers, and one on chromosome 7 (rs1860517). But these associations all mapped to consensus CNVs located in T-cell receptor (TCR) regions (TCR alpha at chromosome 14 and TCR gamma at chromosome 7; Table A in S1 File), and such associations likely reflect somatic, non-inherited alterations rather than association with the SA outcome (see Discussion) [32, 34, 44, 45]. Fig A in S1 File depicts the quantiles vs quantiles (Q-Q) plots of the FBAT-CNV *P*-values for the 88,450 CNV markers, with or without the TCR regions included. There were no significant FBAT-CNV signals remaining after excluding the markers located in the TCR regions, even when analyzing subgroups of diagnoses, medication use or gender *post-hoc* (data not shown).

Next, we used PennCNV to call rare (<1%) and large (>100kb) CNVs, the type of CNVs usually implicated in neurodevelopmental disorders. CNV calls in TCR regions were now removed by recommended QC procedures, but we noted that nine parents had non-transmitted CNVs and one offspring had a *de novo* CNV call in the TCR alpha region (Fig B in S1 File).

After CNV calling and QC we observed 385 putative rare CNVs in 225 SA (47% males; Tables B and C in S1 File). We first evaluated the occurrence of certain well characterized and schizophrenia-associated CNVs [38] in our SA, thus investigating the yet unknown similarity with schizophrenia in this regard. Table 2 outline the burden of putative CNVs in SA offspring in 14 such loci among mainly (7 out of 9) non-schizophrenic SA, in comparison to four previously published control samples of schizophrenia CNV-studies: 1.55% of SA offspring were carriers of CNVs located in schizophrenia-associated loci, which was significantly higher than the 0.24–0.92% carriers observed in the control samples (*P* = 0.004; overall *OR* = 2.57 [1.34–4.92] under random effects model; Figs C and D in S1 File). Combined analysis across all loci also yielded an increase for SA offspring (*P* = 5 x 10^−5^; overall *OR* = 6.33 [2.6–15.4] under random effects model; Fig 1), which remained significant even if nullifying the counts observed for the two schizophrenic SA at the WBS and 15q11.2 loci (*P* = 1.3 x 10^−4^ and overall *OR* = 5.77 [2.35–14.1]). For singular loci, the effects were the highest for *NRXN1* and 22q11.2 deletions (*ORs* = 70–205; Table 2) observed in non-schizophrenic SA. We concluded that these CNVs associate with SA in a similar manner as with schizophrenia, suggesting the role of schizophrenia-associated CNVs in certain (non-schizophrenic) SA subjects.

We then explored all additional CNVs calls with size >1 MB in SA offspring (Table 3), as these are detected with high reliability and may have higher pathological impact than smaller sized CNVs. Six out of 15 CNVs were in regions previously implicated in a CNV-driven pathology, and remaining nine CNVs also affected neural genes of putative interest (Table 3, Fig E in S1 File and Discussion). When including the three additional >1 MB CNVs from Table 2 (WBS dup and 22q11.2 del), there was a total of 18 SA offspring carriers of >1 MB sized CNVs (~3.1%), but this was not significantly higher compared to the rates in three previously published control samples (1.8–3.1%; data not shown) [46–48].

**Table 3:** Additional rare and very large (>1Mb) CNVs observed in 15 SA offspring.

Chr#: start–stop	Size (Mb)	Type	Origin	SA sex	Proximal genes of interest	Pathological CNV?
chr2:198874627–199903114	1.0	Dup	Maternal	*m*	SATB2*	2q33.1 del^1^
chr3:57010–1054024	1.0	Dup	Maternal	*f*	CHL1*, CNTN6	*ASD*^2^
chr3:1134787–2375967	1.2	Dup	Maternal	*f*	CNTN6*, CNTN4*	*ASD*^2^
chr4:31014644–32191478	1.2	Dup	Maternal	*m*	*PCDH7*	
chr4:75528333–76702535	1.2	Dup	Paternal	*m*	AREG*, BTC*	
chr4:188342786–189950157	1.6	Dup	Maternal	*m* ^P^	ZFP42*	
chr4:188698113–189920157	1.2	Dup	Paternal	*m*	ZFP42*	
chr6:154918196–155991693	1.1	Del	*De novo*	*f* ^M^	TIAM2*	
chr8:57437447–59006759	1.6	Del	Paternal	*m*	PENK*	
chr11:79782071–81358036	1.6	Dup	Paternal	*f* ^M^	*ODZ4*, *DLG2*	
chr12:56820511–59741054	2.9	Del	Paternal	*f*	LRIG3*	
chr13:46505958–47784283	1.3	Dup	Maternal	*m*	RB1*, HTR2A	*RB1*^3^
chr16:15550310–18070334	2.5	Del	Paternal	*m*	NDE1*, ABCC1*	16p11.2^4^
chr17:32227936–33270047	1.0	Dup	*De novo*	*f* ^P^	LHX1*, many	17q12 del^4^
chr18:10587874–11910658	1.3	Dup	*De novo*	*m*	FAM32B*, GNAL*	

Positions are in the NCBI36/hg18 assembly. See Fig E in S1 File for plots of genomic locations and LRR / BAF profiles. Del, deletion; Dup, duplication; SA, suicide attempter; *m*, male; *f*, female.

^1^ Reviewed in ref [28].

^2 ^*CHL1* and *CNTN4* CNVs in autism spectrum disorders (ASD) [28].

^3^ Reviewed in ref [28].

^4^ Described also in Table 2 and reviewed in ref [28].

* Gene intersect with CNV boundary

^M^ SA diagnosed with MDD

^P^ SA diagnosed with PTSD

We sought to identify medically relevant genes previously shown affected by CNVs (among those summarized by Zarrei *et* al. [28]). In total, there were 752 medically relevant and CNV-affected genes possible to find in our genomic CNV to gene mapping, which defined the genomic background rate (752/19897 = 3.8% of all genes). Among the 385 CNV calls in SA offspring which intersected 675 genes in total, we observed 65 of the medically-relevant genes (observed rate of 65/675 = 9.6%), which represented a significant enrichment when compared to the genomic background rate (*P* = 4 x 10^−12^ by hypergeometric test). Sensitivity analysis showed that this enrichment remained at *P*<0.05 even if one removed up to 30 from the 65 genes (Table D in S1 File) observed here. There were 45 SA carriers (58% males) of 47 CNVs that intersected these 65 genes (Table D in S1 File), which included all CNVs in schizophrenia-associated loci (listed in Table 2), six of the additional >1MB CNVs (Table 3) as well as 28 other CNVs and SA carriers (Table 4). A PPI network of these 65 genes is shown in Fig F in S1 File, wherein the 49 observed connections were significantly more than the 8 expected by chance (according to the STRING website), indicating a shared biological function. Indeed, all the significant gene set enrichments of Gene Ontology biological processes observed were related to neurodevelopmental or neurogenesis functions (with 29 out of 65 genes involved; Table E in S1 File).

**Table 4:** Additional putative CNVs which intersected with medically relevant genes, previously shown affected by CNVs.

Chr#: start–stop	Size (Kb)	Type	Origin	SA sex	Medically relevant, CNV-affected gene(s) ^1^
chr1:229774037–229879757	105.7	Dup	Paternal	*f*	*DISC1*
chr2:31452327–31659330	207.0	Dup	Maternal	*m* ^S^	*SRD5A2*
chr2:161972461–162073448	101.0	Del	Maternal	*m* ^M^	*SLC4A10*
chr2:176643771–176756047	112.3	Del	Paternal	*m* ^M^	*EVX2*, *HOXD13*
chr3:2899986–3102519	202.5	Dup	Maternal	*m*	*CNTN4*
chr5:150082437–150312674	230.2	Dup	*De Novo*	*m* ^M^	*IRGM*
chr5:150156524–150260690	104.2	Dup	Paternal	*m*	*IRGM*
chr7:2527303–2659332	132.0	Del	*De Novo*	*m* ^S^^,^^A^	*LFNG*
chr9:21718683–21847303	128.6	Dup	Paternal	*m* ^M^^,^^A^	*MTAP*
chr9:28603702–28835945	232.2	Del	Maternal	*m* ^P^	*LINGO2*
chr9:28652170–28837621	185.5	Del	Maternal	*f* ^S^	*LINGO2*
chr9:93269250–93470947	201.7	Dup	Maternal	*f* ^A^	*ROR2*
chr11:706765–836070	129.3	Dup	Mat/Pat	*m*	*SLC25A22*
chr12:118727882–118912851	185.0	Del	*De Novo*	*m* ^P^	*CIT*
chr15:97271086–97743963	472.9	Dup	Maternal	*m*	*IGF1R*
chr16:21856623–22328822	472.2	Del	Maternal	*f* ^M^	*POLR3E*, *EEF2K*, *CDR2*
chr16:21856623–22298757	442.1	Dup	*De Novo*	*f*	*POLR3E*, *EEF2K*, *CDR2*
chr17:1015143–1217312	202.2	Dup	*De Novo*	*f*	*BHLHA9*, *YWHAE*
chr17:31559291–31810869	251.6	Del	Paternal	*m*	*CCL4L1*, *CCL4L2*, *CCL3L1*
chr17:31559291–31699832	140.5	Del	*De Novo*	*f*	*CCL4L1*, *CCL4L2*, *CCL3L1*
chr17:31621324–31822399	201.1	Del	*De Novo*	*m* ^S^	*CCL4L2*, *CCL3L1*
chr19:2172792–2279281	106.5	Dup	*De Novo*	*m* ^M^	*AMH*
chr19:3924600–4053449	128.8	Dup	Maternal	*f*	*MAP2K2*
chr19:3924600–4053449	128.8	Dup	Maternal	*f*	*MAP2K2*
chr21:34625589–34783587	158.0	Del	Paternal	*m* ^P^	*KCNE2*
chr21:36406812–36514611	107.8	Dup	Maternal	*m* ^M^	*KCNE1*, *DOPEY2*
chr21:36406932–36514611	107.7	Dup	Maternal	*f* ^P^	*DOPEY2*
chr22:32194817–32314329	119.5	Del	Maternal	*f*	*LARGE*

Positions are in the NCBI36/hg18 assembly. See also Table D in S1 File. Del, deletion; Dup, duplication; SA, suicide attempter; *m*, male; *f*, female.

^1^ As summarized in ref [28].

^S^ SA diagnosed with schizophrenia

^M^ SA diagnosed with MDD

^P^ SA diagnosed with PTSD

^A^ SA used psychotropic medications prior to the index SA

*De novo* CNVs may have increased pathogenic potential compared to inherited CNVs. Overall, 26% (100 of 385) of SA offspring CNV calls were *de novo*, and 12% were not found in any parent at all. For the schizophrenia-associated loci in Table 2, the rate increased to 67% *de novo*, and 44% not found in any parent (*NRXN1* del, WBS dup and 22q11.2 del). Overall, 54 of SA (9.3%) were *de novo* carriers (59% males; Table C in S1 File), which is comparable to the high rates of ~3–7% previously reported for schizophrenia [49]. But neither diagnosis, gender nor medication use (Table 1) were found overrepresented in SA CNV carriers (*de novo* or all) compared to SA CNV non-carriers (*P* > 0.05; data not shown), suggesting that these secondary outcomes did not explain the CNV-rates among SA herein.

Finally, we compared the CNV calls with our recently published SNP-based GWAS, which showed an increase of common SNP risk-allele burden (polygenic risk) among neurodevelopmental genes in SA [27]. Among the 675 genes intersected here by CNVs in SA offspring, 35 belonged to the same neurodevelopmental gene set that we had used in the GWAS (*P* = 0.033 by hypergeometric test), wherein 17 genes involved both CNV calls and contributed to common SNP risk-allele burden in SA (e.g. *NRXN1* and *CNTN4*; Table F in S1 File). We therefore repeated the polygenic association of our previous GWAS [27], now testing SA CNV carriers and CNV non-carriers as ‘genetic subgroups’. However, common SNP risk-alleles explained 0% of the variation in SA CNV carriers, while increasing to 7.8% (*P* = 0.001) of variation explained in SA CNV non-carriers (the original result in all SA was 4.9% [27]). To control for the sample size differences between carriers and non-carriers, we also re-analyzed ten random draws of n = 116 CNV non-carriers (to have the same sample size as CNV carriers); the variation explained ranged between 1–13% (mean = 8.7%, 95% CI 5.9–11.5% and Shapiro–Wilk test *P* = 0.9) and the 0% observed for CNV carriers was thus significantly lower (*P* = 6.2 x 10^−5^). We concluded that SA CNV carriers may form a ‘genetic subgroup’ with regard these neurodevelopmental genes, since they contributed less to the SNP risk-allele burden than CNV non-carriers [27].

## Discussion

We first observed significant FBAT-CNV associations in SA, but they all mapped to T-cell receptor (TCR) regions. A multitude of somatic TCR gene rearrangements normally occurs during lymphocyte development, generating the polyclonal blood cell population which has the required variability to recognize all different antigens. Furthermore, the proportion of lymphocytes (and their DNA) may also vary between individuals in a state-dependent manner. Together, this produces variable signal intensities at these TCR loci which may complicate e.g. FBAT-CNV associations. As our source of DNA was blood, the TCR-associations observed here likely reflect inter-individual variation in somatic rearrangements and/or proportions of lymphocytes, rather than association with SA [32, 44]. This problem has been highlighted by others previously, e.g. comparing CNV-associations between different sources of DNA and performing confirmatory PCRs [44]. Furthermore, it has also been previously reported about spurious CNV-associations of TCRs on chromosome 14 and 7 when using the FBAT-CNV methodology, involving age-dependent effects with regard to signal intensity in TCRs [34]. It is indeed a common practice to remove recombination regions such as TCRs as part of QC when calling CNVs using e.g. PennCNV. In conclusion, the FBAT-CNV methodology did not detect any CNVs of interest for future follow-up, and suggested no role of common CNVs with moderate effects (*OR* > 1.6) on SA outcome.

But after calling rare and large CNVs using the PennCNV algorithm in SA offspring, we observed the occurrence of CNV-regions and genes which have previously been implicated in neurodevelopmental disorders, both in schizophrenia (Fig 1) and other psychopathologies. This was a novel finding, since the previous case-control study of CNVs in SB did not report about any such pathogenic CNV candidates in SB [15]. Overall, we observed the enrichment of 65 medically relevant and CNV-affected genes in 45 SA CNV-carriers (Table D in S1 File), which could be of particular interest to consider in future follow-up studies. These 65 genes had enrichment of neurodevelopmental and neurogenesis biological functions (Table E in S1 File). This biology was congruent with our recent GWAS, concerning a SNP-based polygenic risk among neurodevelopmental genes in SA [27]. Interestingly, our SA CNV-carriers contributed less to this SNP-based risk than CNV non-carriers, suggesting that genetic variation in neurodevelopmental genes that impact SA risk may be driven by either common SNPs or rare CNVs, in different SA subjects. In contrast, it was recently shown that schizophrenic carriers of rare CNVs (having neurodevelopmental effects) also had an overall increase in the SNP-based risk [50].

Some of the rare SA CNV calls intersected with genes previously implicated by others in SB. *COMT* and *DGCR8* were *de novo* deleted in two SA females in the schizophrenia-associated 22q11.2 CNV (Table 2), but *COMT* is also one of the earliest SB candidate genes [51] with shown SNP-association and brain expression alterations, and *DGCR8* also showed SNP-association [8]. We also observed CNVs affected other previous SB candidate genes e.g. *DISC1*, *LSAMP*, *YWHAE*, *TMEM132C*, *CD300LB* and *HTR2A* (Tables 3 and 4) [8]. However, the majority of neurodevelopmental genes implicated here by CNVs have not been implicated previously in SB, except in our previous SNP-based GWAS (see Table F in S1 File) [27]. Together, this CNV study and our previous SNP-based GWAS suggest a number of novel neurodevelopmental and neurogenesis candidate genes of putative interest for future studies, with regard to either their SNP or CNV genetic variation. At present we can only speculate about any specific molecular mechanism, but from viewing the enriched biological processes (Table E in S1 File) it’s obvious that early development and neurogenesis processes may be involved. While the role of neurogenesis throughout life has significant focus in SB research as related to e.g. epigenetics and early-life adversity [5], less is known about neurodevelopmental processes and genes acting prenatally. Interestingly, the importance of the prenatal period was suggested by the association of restricted fetal growth with SB in adulthood [52]. Molecular evidence for prenatal effects in SB is scarce, but recent observation report about a low digit ratio (2D:4D) in male suicide victims, a trait which is a proxy for the prenatal exposure to androgens [53]. While the androgen receptor (*AR*) gene was not directly observed among our CNVs *per se*, *AR* has “guilt-by-association” in the PPI network presented here due to its central connectivity to the *IGF1R*, *NDE1*, *RB1* and *AMH* genes therein (if inserted into Fig F in S1 File; not shown), which is of interest to consider for future studies.

This study has several limitations. The FBAT-CNV method may be sensitive to non-linear relationships between raw intensity values and CNV genotypes [54] and we had limited power to detect CNV-effects (*OR* < 1.6). Despite using trios to validate inherited CNV calls from PennCNV and applying extensive QC procedures, we did not confirm any CNVs with PCR, whereby a fraction of false-positive CNVs is to be expected (5–10% false positives). In this regard, the selected schizophrenia-associated CNVs, large >1MB CNVs and CNVs overlapping medically relevant genes previously shown affect by CNVs, should have higher likelihood to be valid and useful for interpretations and follow-up studies (compared to e.g. completely novel CNVs). Finally, we lacked our own non-SA controls (optimally being unaffected sib’s with matched psychiatric disorder status), which hampered our possibilities to robustly test for association and linkage with SA outcome *per se*. For example, while it was of interest to observe schizophrenia-associated CNVs in non-schizophrenic SA, this at best reflects an overlap between schizophrenia and SA rather than a specific association with SA. Taken together, the rare CNVs presented here for SA are to be regarded as preliminary observations which require future confirmation. Finally, the rare CNVs indicated in this study are only relevant for a minor subset of SA subjects; overall ~7% (*n* = 45) of SA had CNVs which affect medically relevant genes.

Previous “traditional” SNP-based GWAS have failed to observe any consistent genome-wide significant associations with SA [12–14, 55]. It is now increasingly clear that the overall genetic etiology of SB is most likely polygenic, involving many genes and genetic variants of small effects across several neurosystems [6–10]. Up to date, two published GWAS on SB outcomes used a polygenic risk scoring methodology and thereby observed modestly significant overlap of the common SNP risk-allele burden with schizophrenia or major depression [10, 27]. We showed that overlap with schizophrenia involved neurodevelopmental genes, but not e.g. the schizophrenia-implicated MHC-region [27]. Rare CNVs are also of clear importance in schizophrenia, involving a neurodevelopmental component [16] and we here observed overlap with SA regarding such CNVs as well. Rare and large CNVs with developmental impact can be expected to have pleiotropic effects with regard to psychiatric outcomes in later life [56]. Indeed, other diagnoses than schizophrenia which are usually not implicated with rare CNVs were prevalent in our SA CNV carriers (depression and PTSD), and many SA CNV carriers did not fulfill any diagnostic criteria. Such pleiotropy in psychiatric outcomes driven by rare CNVs is also congruent with the transdiagnostic dimension shown in SB (consisting of a shared effect among all mental disorders)[57]. Taken together, our preliminary observations indicate a role of rare pathogenic CNVs affecting neurodevelopmental functions in a subset of SA, who were distinct from SA having increased SNP risk-allele burden. These observations may open up new avenues in the genetic etiology of SB.

## Supporting Information

S1 File Supplementary Figs A-F and Tables A-F.
(PDF)

S1 Table Output from FBAT-CNV analysis of CNV-loci. Marker, the HumanOmni1-Quad_v1 CNV marker ID; Position, according to NCBI 36.3 assembly.
(XLSX)

S2 Table Output from FBAT-CNV analysis of SNP-loci. Marker, the HumanOmni1-Quad_v1 SNP ID; Position, according to NCBI 36.3 assembly.
(XLSX)
