# Supplementary material for: Rare CNVs in Suicide Attempt include Schizophrenia-Associated Loci and Neurodevelopmental Genes: A Pilot Genome-Wide and Family-Based Study
Source: PLoS One. 2016 Dec 28;11(12):e0168531. doi: 10.1371/journal.pone.0168531 (PMC5193342; doi:10.1371/journal.pone.0168531)
Supplement: S1 File — Supplementary Figs A-F and Tables A-F. (PDF) [file pone.0168531.s001.pdf]

A

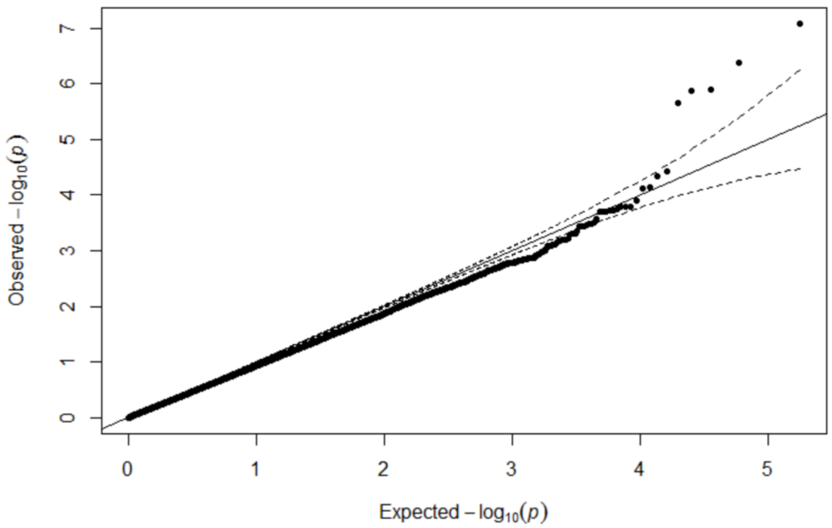

B

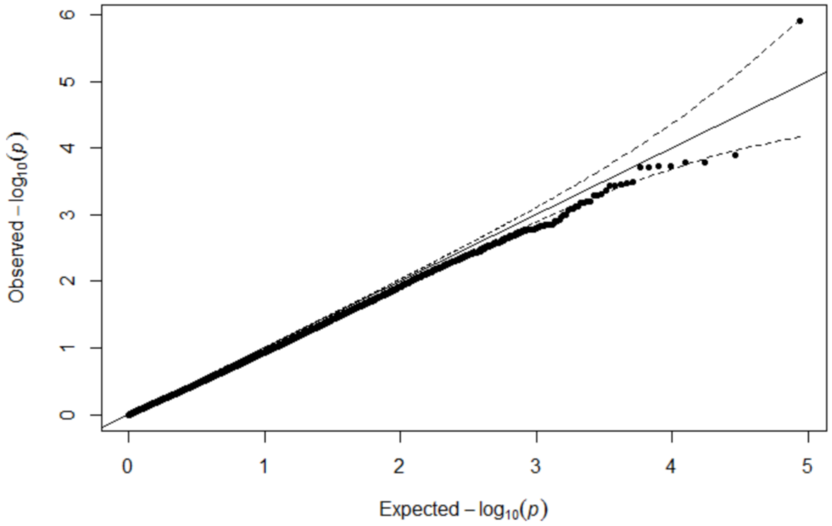

Fig A. Q-Q plots of FBAT-CNV *P*-values testing the association and linkage among 88,450 genome-wide CNV markers, with and without the T-cell receptor regions. Dotted line show the 95% confidence intervals of the expected –log(*p*) uniform (0,1) scale. The inflation factor λ is the ratio of observed median –log(*p*) *vs* expected median –log(*p*). (A) Q-Q plot including T-cell receptor regions; λ = 0.94. (B) Q-Q plot excluding CNV markers in T-cell receptor regions alpha and gamma; λ = 0.92.

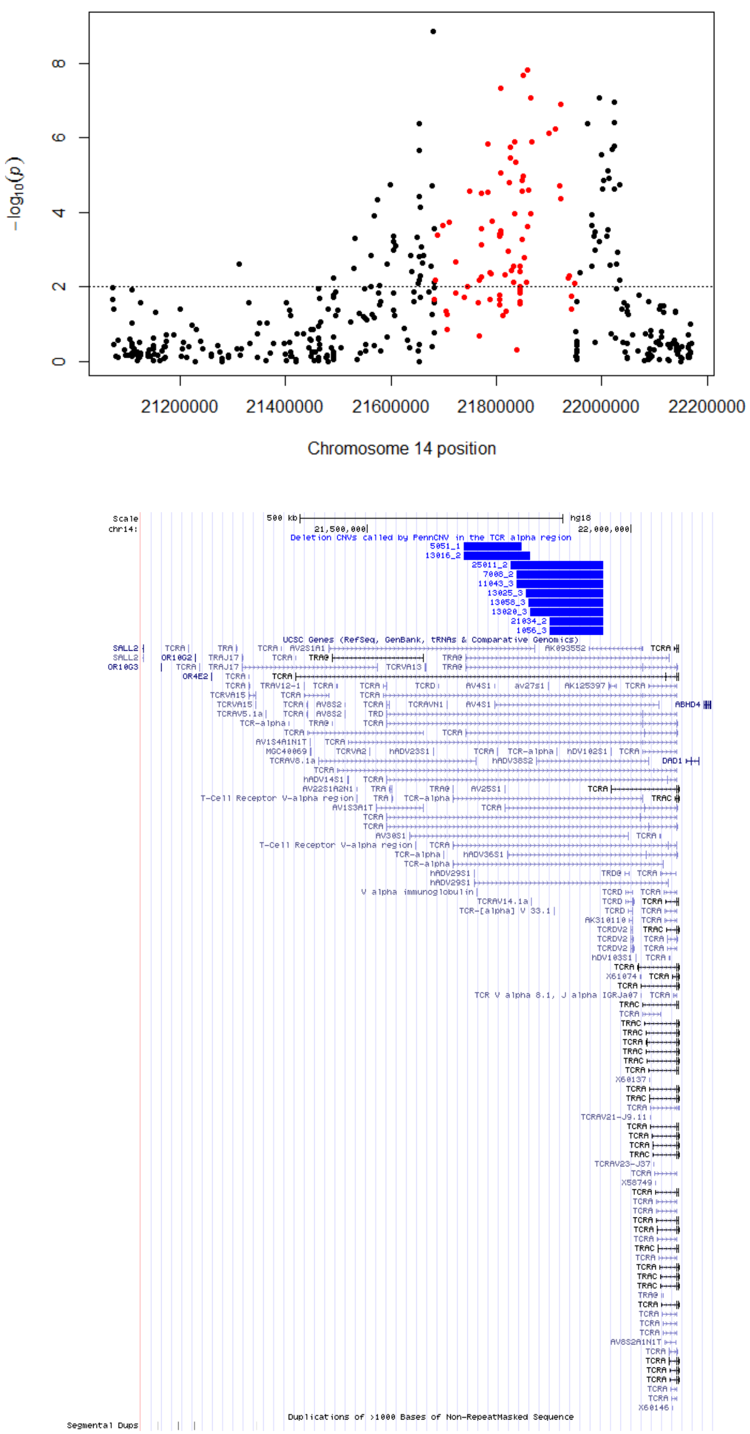

Fig B. Rare and large CNVs in the T-cell receptor alpha region called by PennCNV. Several non-transmitted CNV calls coincided with the low FBAT-CNV P-values (blue bars and red dots delineate same genomic region).

Fig C. Putative pathogenic CNVs in SA offspring. Genomic locations (upper panel) and LRR / BAF profile plots (lower panels) are shown. Positions are in the NCBI36/hg18 assembly.

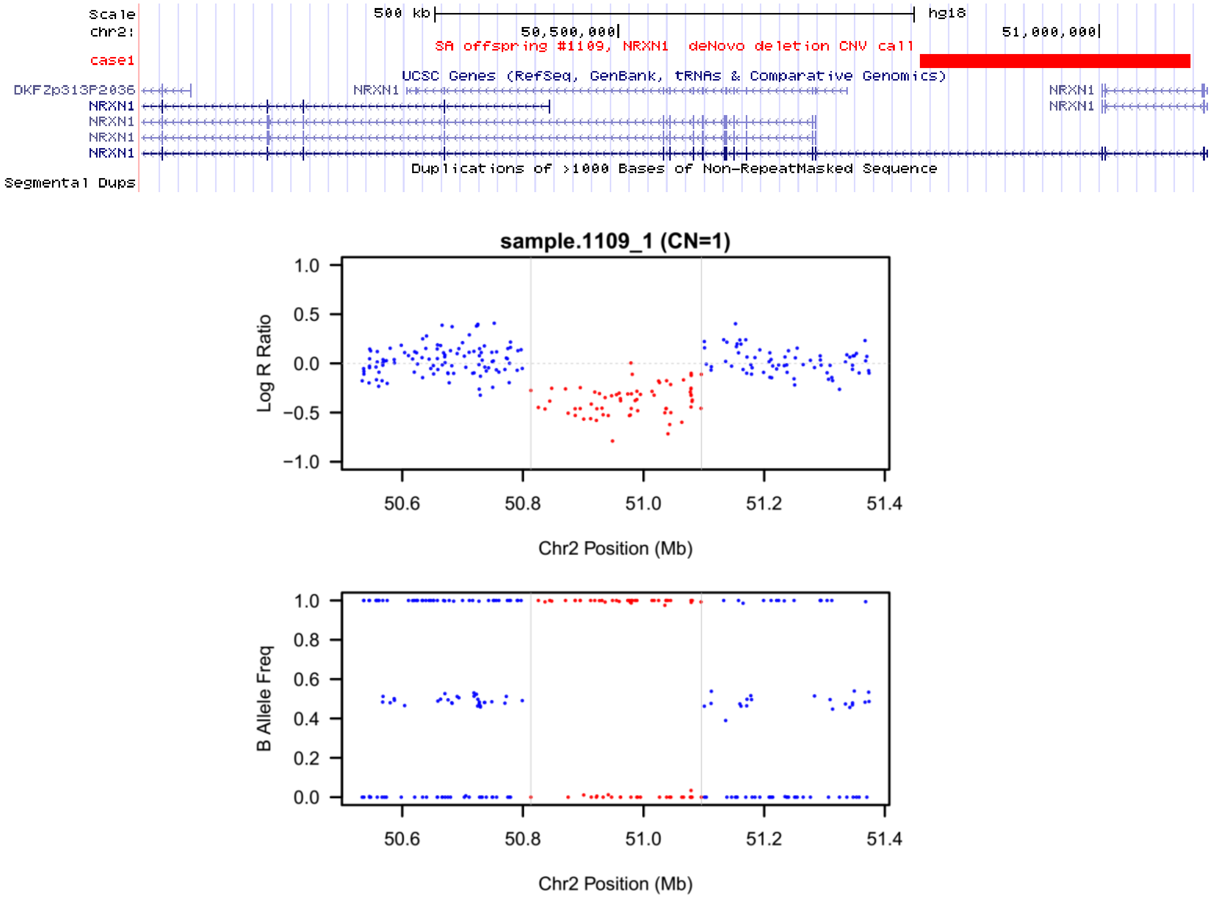

Fig C. (A) *NRXN1 de novo* deletion (red bar) observed in 1 SA offspring .

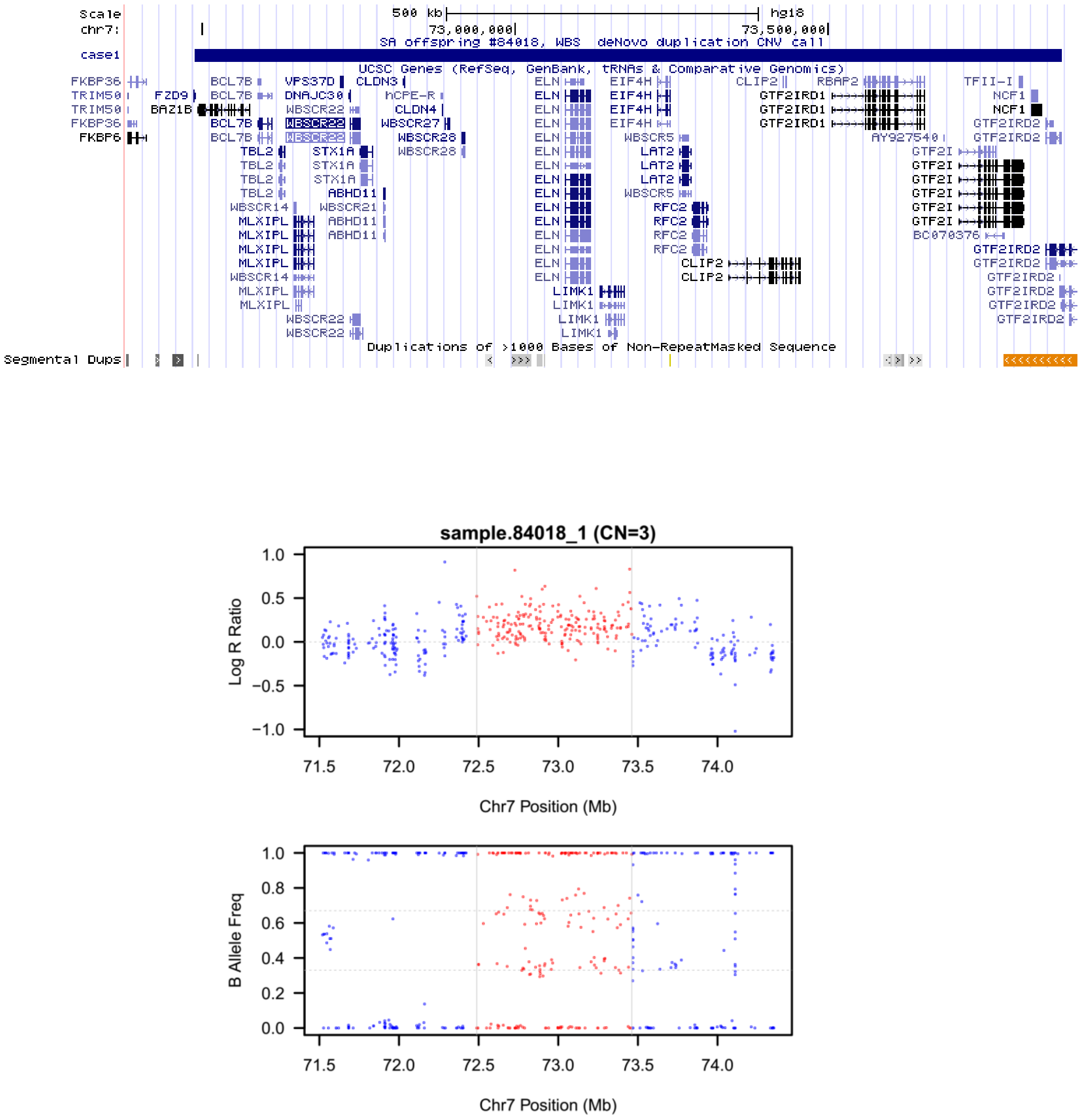

Fig C. (B) Williams-Beuren syndrome (WBS) *de novo* duplication (blue bar) observed in 1 SA offspring, whom was also diagnosed with Schizophrenia.

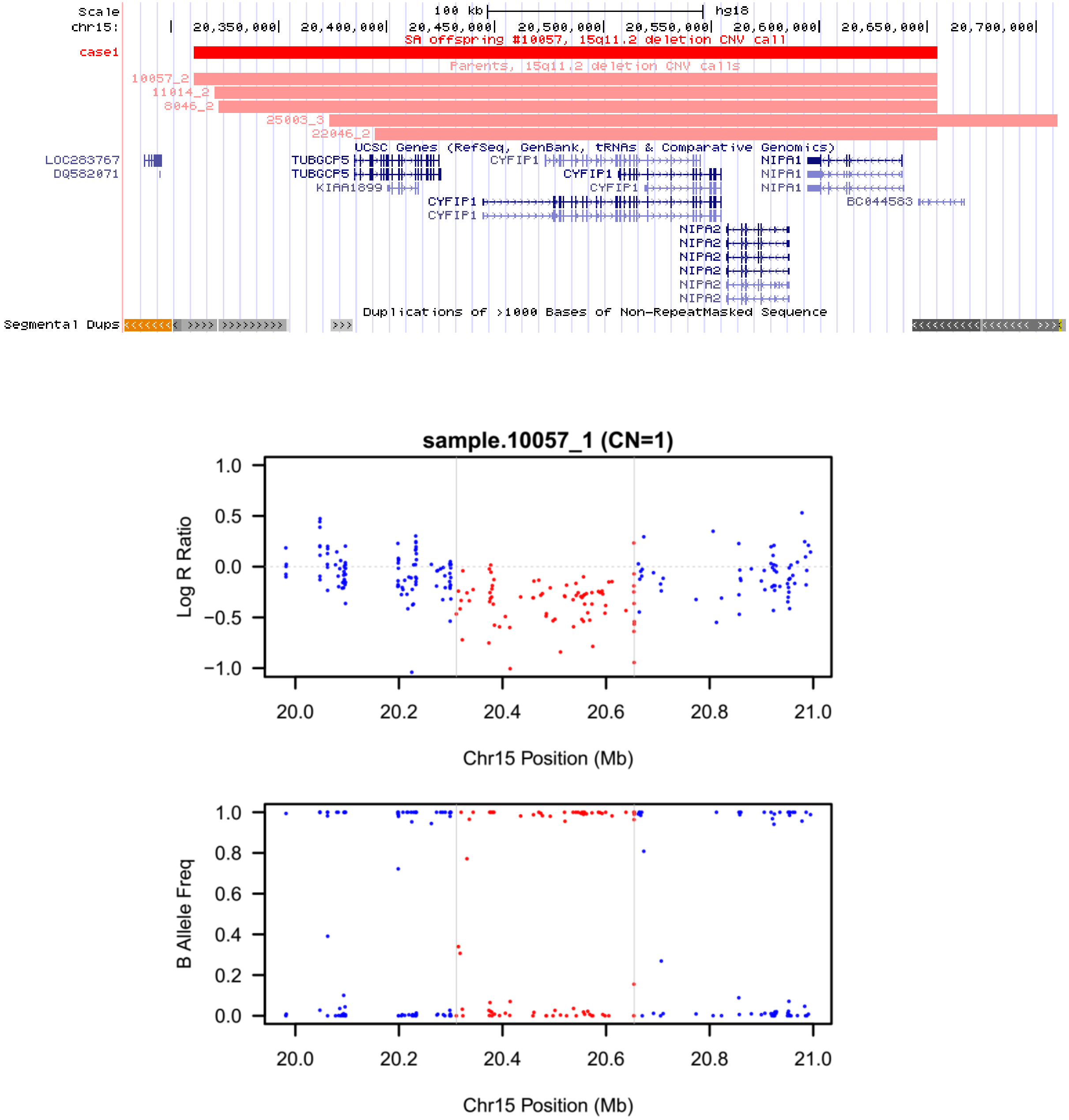

Fig C. (C) A 15q11.2 maternally inherited deletion observed in 1 SA offspring (red bar), whom was also diagnosed with Schizophrenia. The locations of 15q11.2 deletions observed in 5 parents are also shown (pink bars).

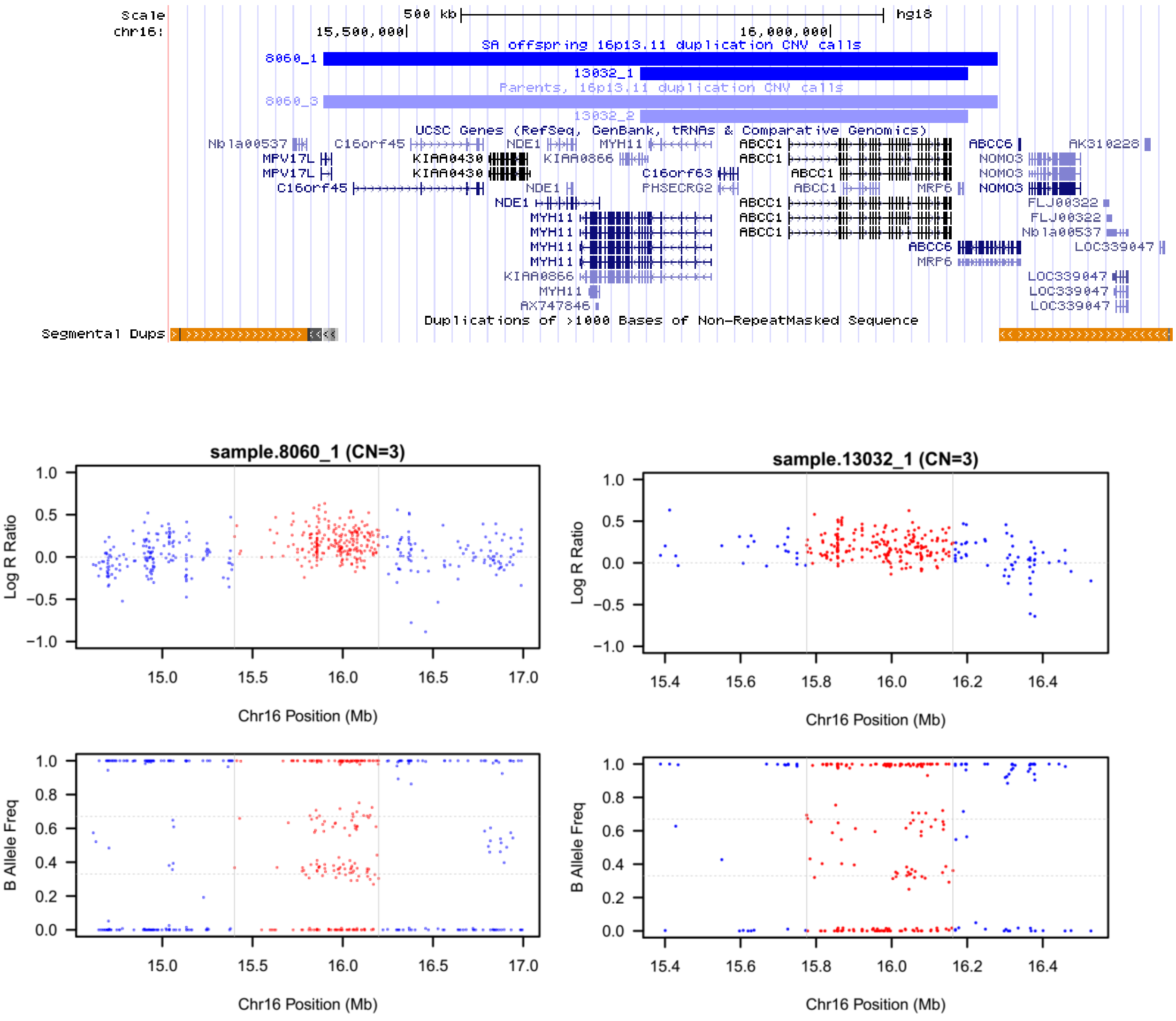

Fig C. (D) Inherited 16p13.11 duplications observed in 2 SA offspring (blue bar). The locations of the 16p13.11 duplications in the 2 parents (one mother and one father) are also shown (light blue bars).

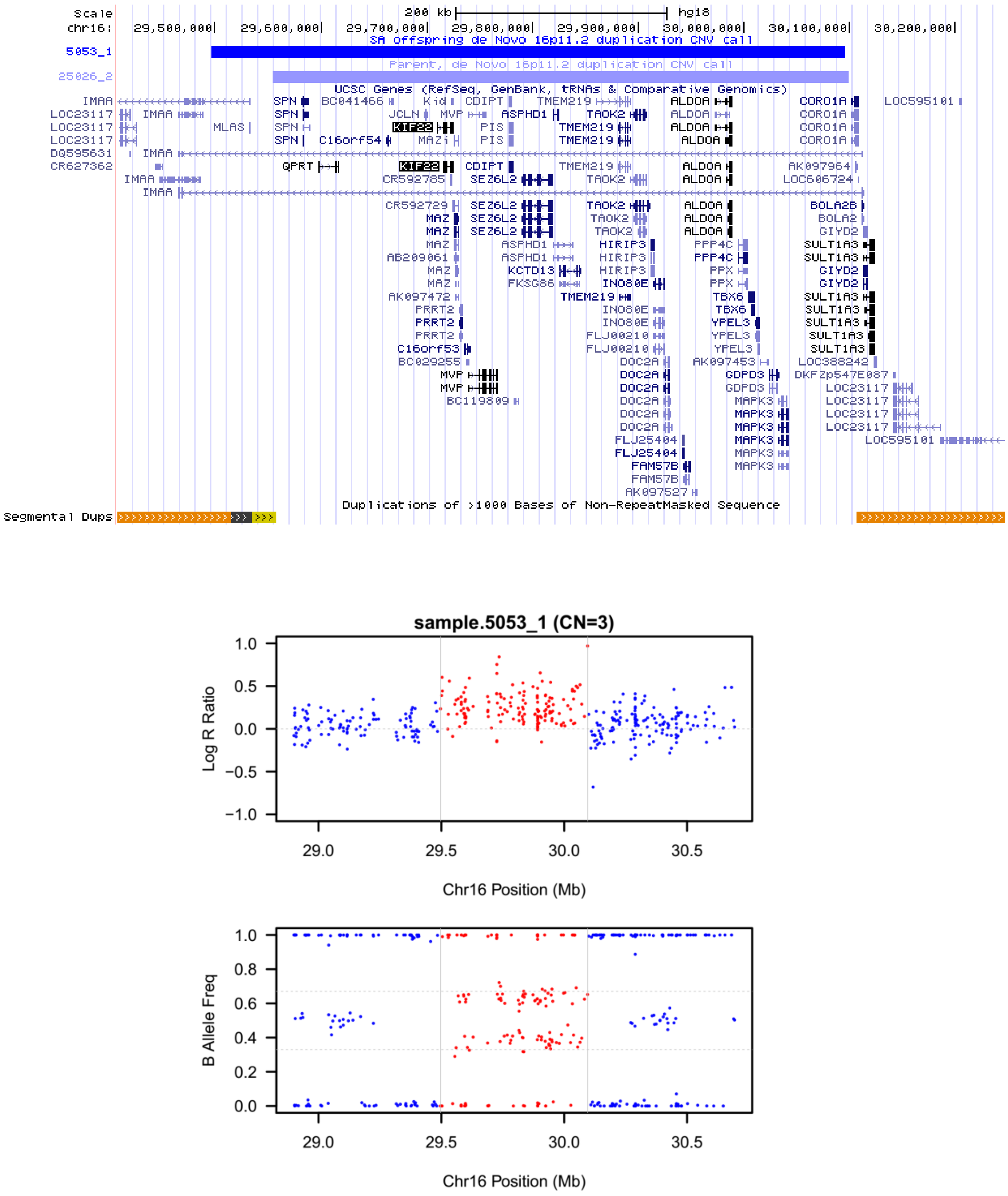

Fig C. (E) 16p11.2 *de novo* duplication observed in 1 SA offspring (blue bar). An unrelated parent also had this duplication (light blue bar).

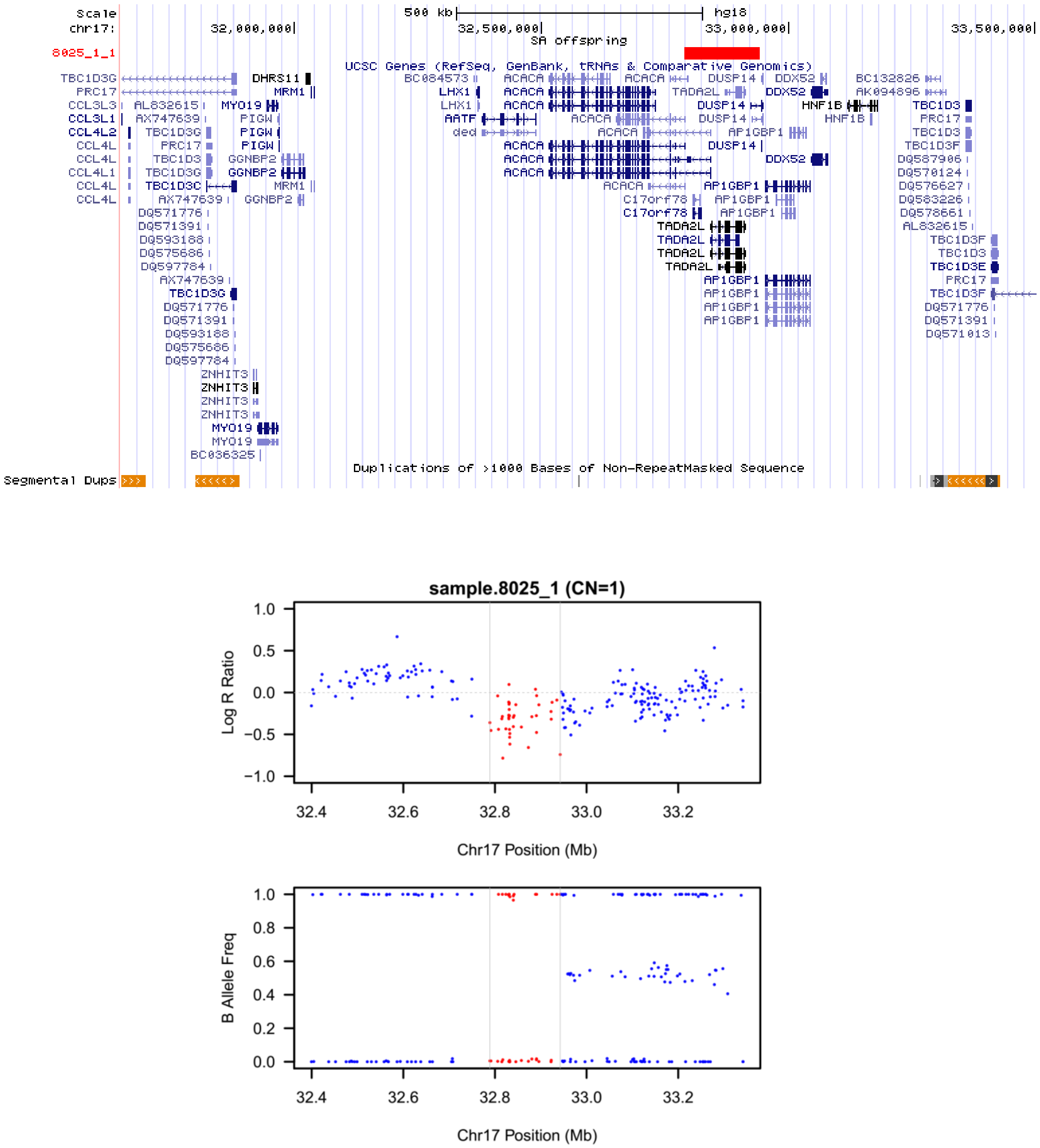

Fig C. (F) A 17q12 *de novo* deletion (130kb) called in 1 SA offspring (red bar). A larger >1MB *de novo duplication* was also observed in another SA.

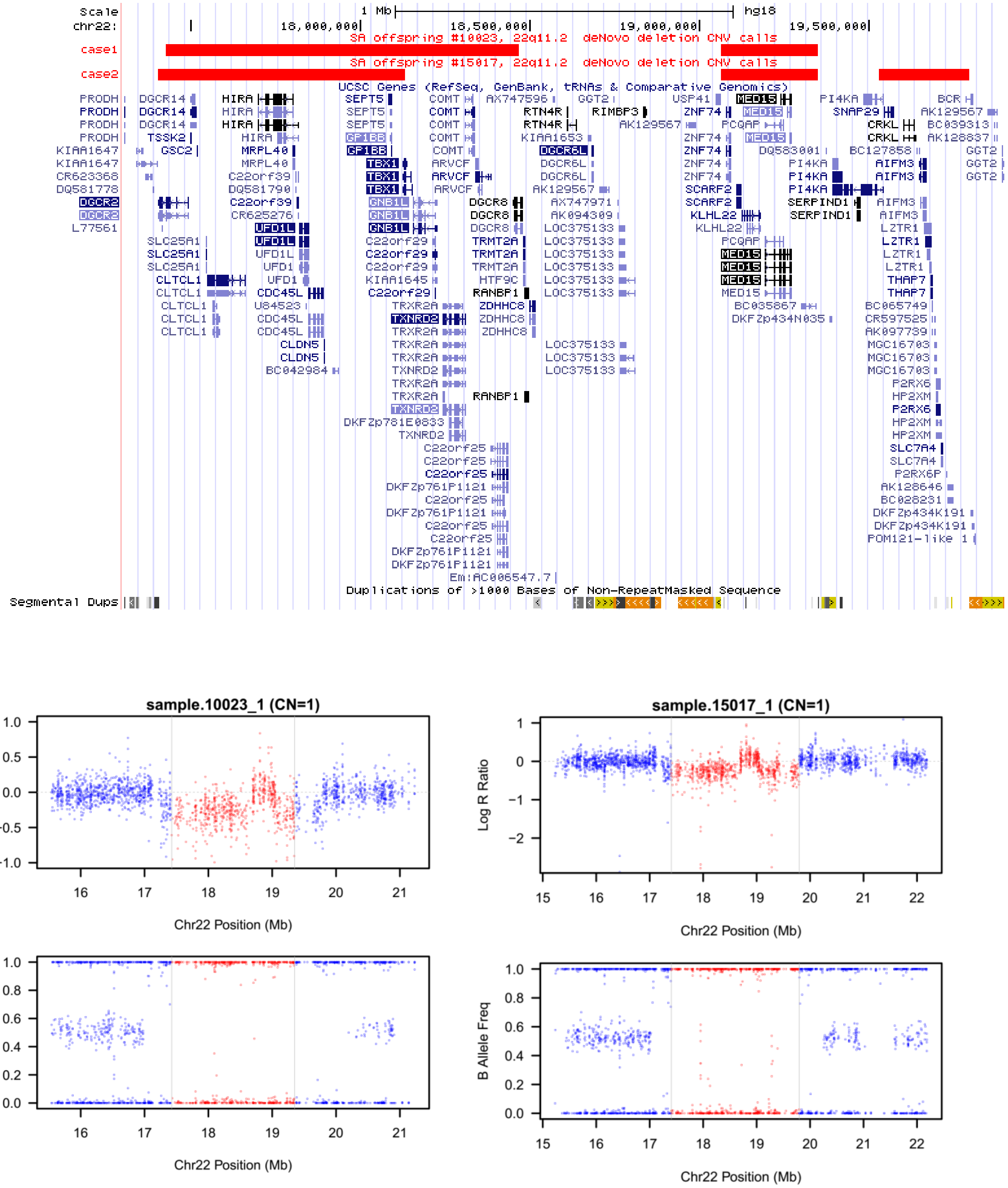

Fig C. (G) 22q11.2 *de novo* deletions (red bars) observed in 2 SA offspring, in-between known segmental duplications (potential recombination breakpoints for CNVs).

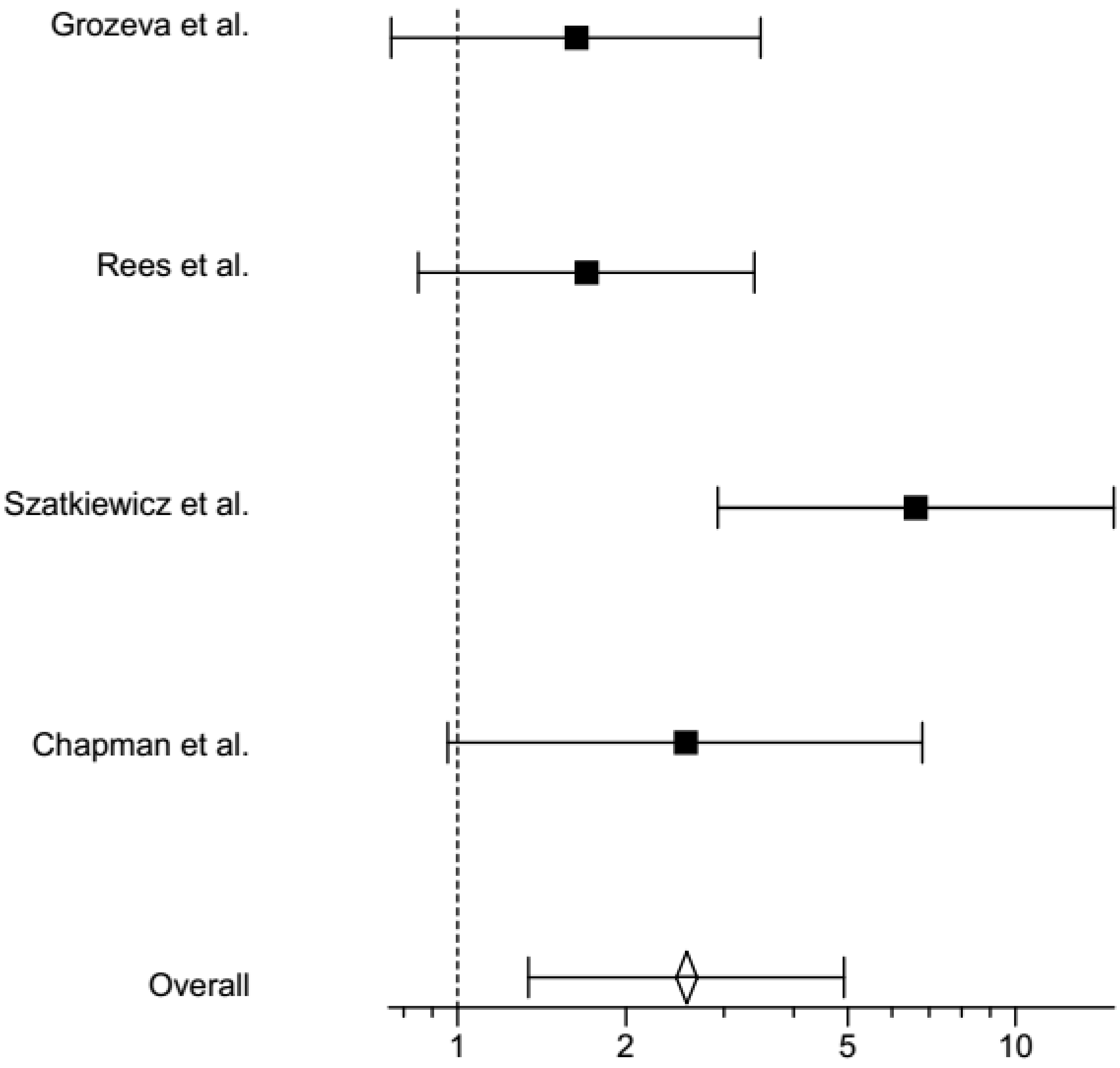

Fig D. Odds ratios (95% CI) for burden of putative pathogenic CNVs in SA offspring, as compared to the rates observed in 4 previously published control samples [1-4]. Overall is a meta-analysis under a random effects model , by help of WinPepi v.3.83 [5].

Fig E. Additional large (≥ 1MB) CNVs calls in SA offspring. Genomic locations (upper panel) and LRR / BAF profile plots (lower panels) are shown. Positions are in the NCBI36/hg18 assembly.

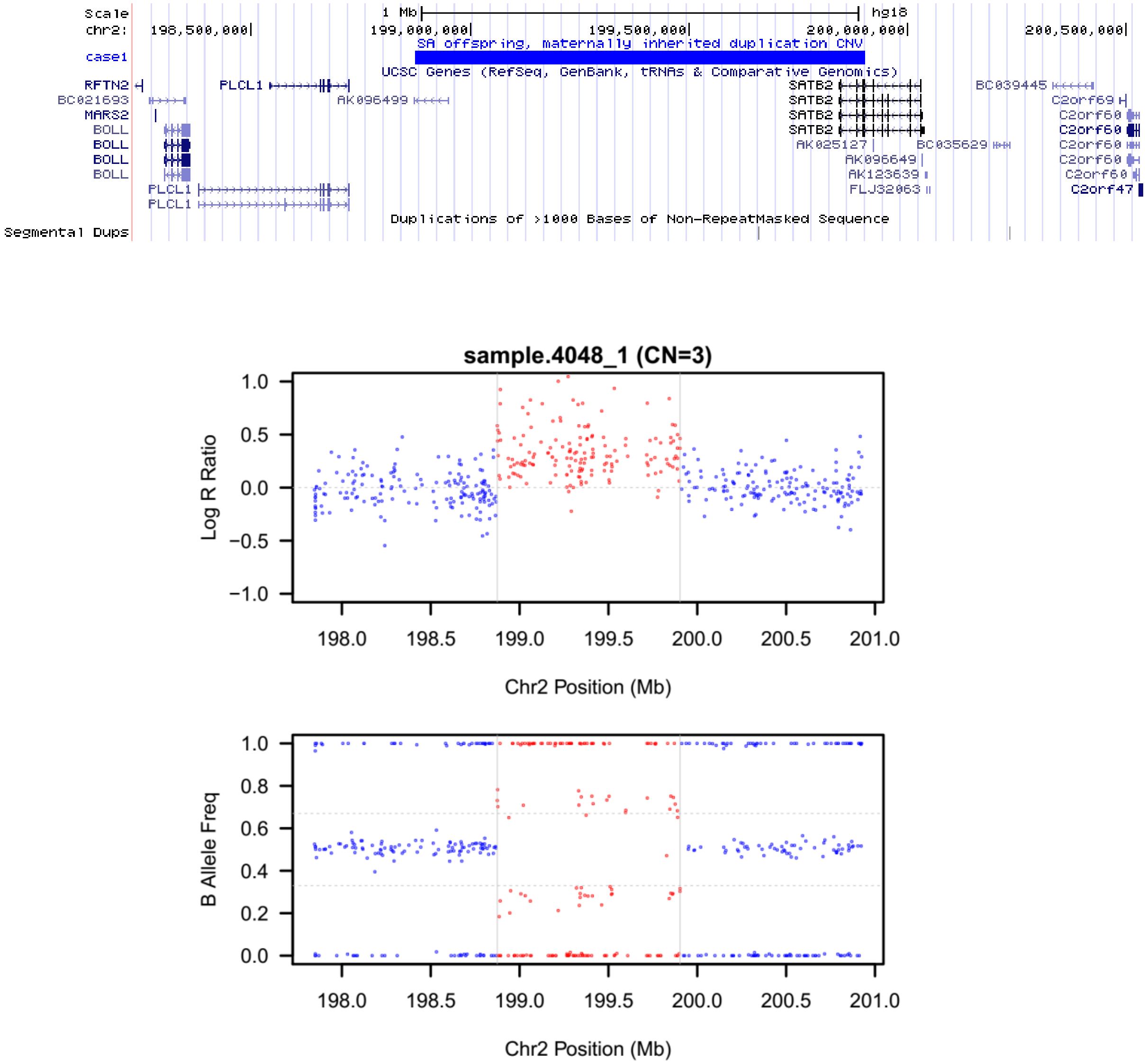

Fig E. (A) A maternally transmitted duplication (blue bar) observed in 1 SA offspring, intersecting the *SATB2* homeobox gene involved in brain development and the 2q33.1 microdeletion syndrome. CNV boundaries (size) are chr2:198874627-199903114 (~1 MB).

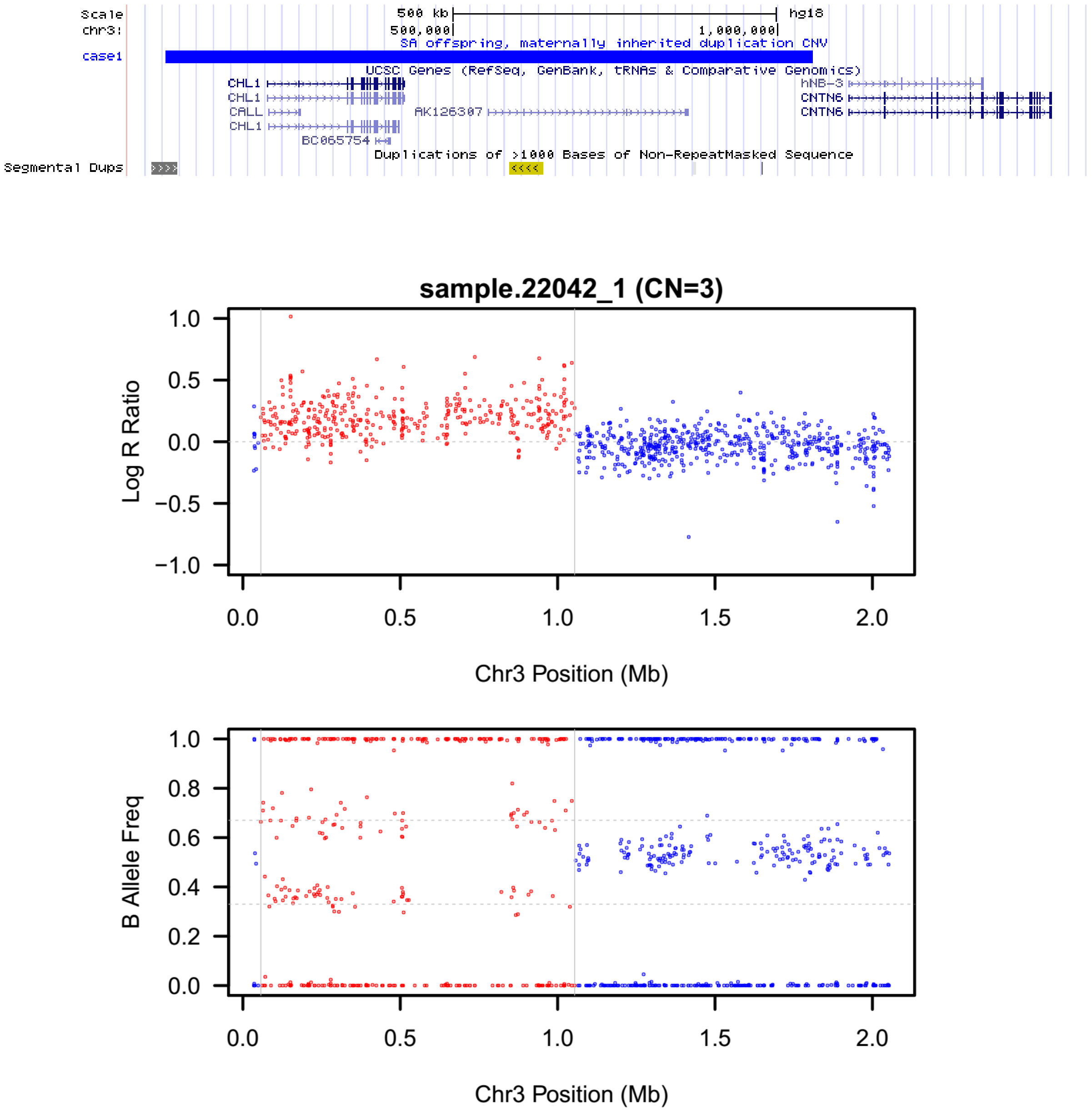

Fig E. (B) A maternally transmitted duplication (blue bar) observed in 1 SA offspring, affecting *CHL1* neural cell adhesion molecule and located directly upstream of *CNTN6*. The deletion of the former is implicated in the mental defects of 3p syndrome, and both genes act as adhesion molecules in the developing nervous system. CNV boundaries (size) are chr3:57010-1054024 (~1 MB).

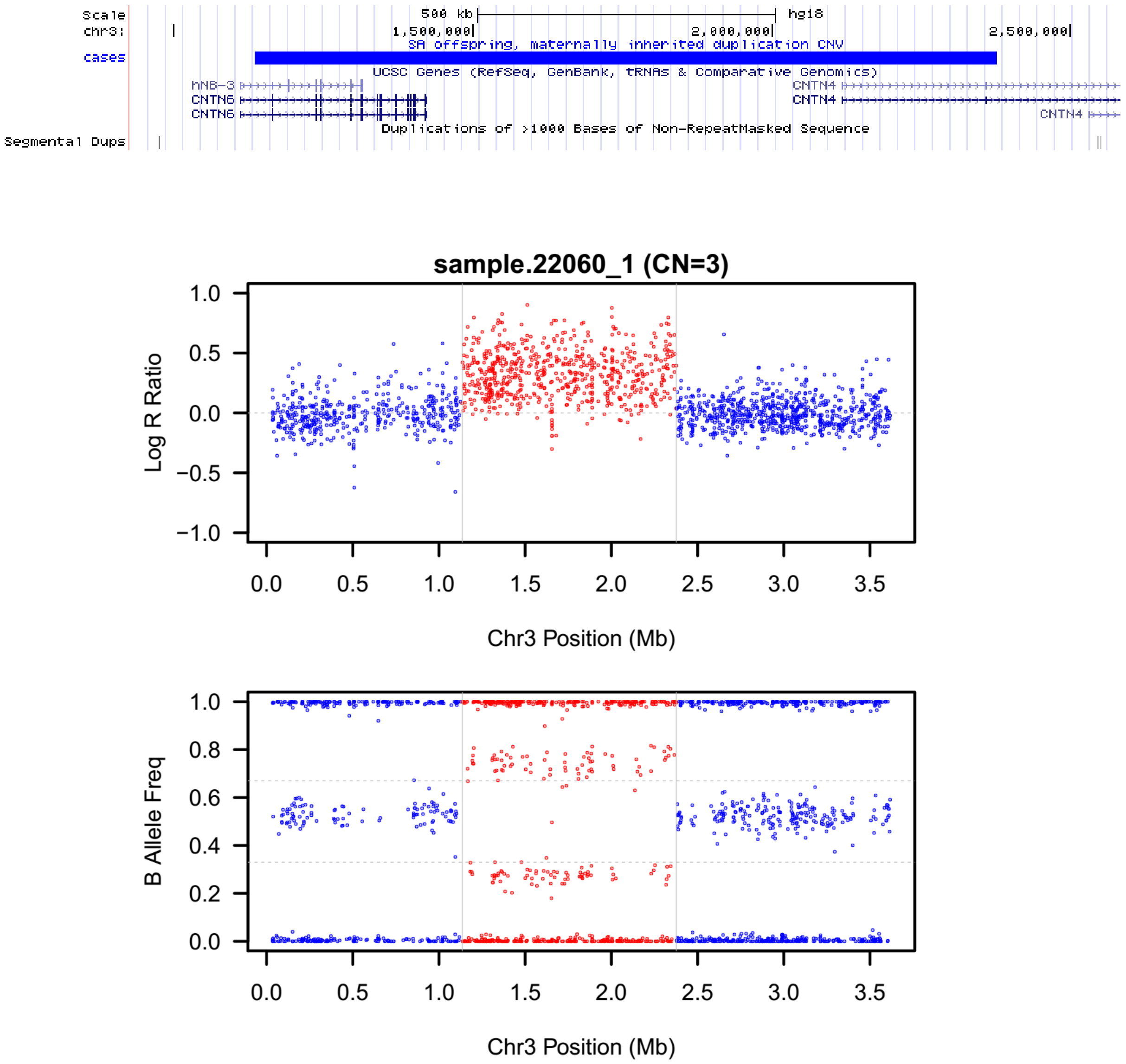

Fig E. (C) A maternally transmitted duplication (blue bar) observed in 1 SA offspring, affecting *CNTN6* and *CNTN4* cell adhesion molecules which are involved in the developing nervous system. CNV boundaries (size) are chr3:1134787-2375967 (~1.2 MB).

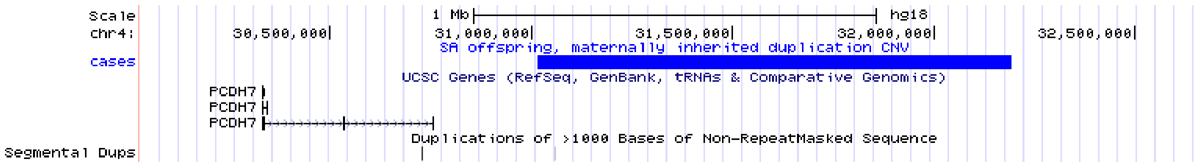

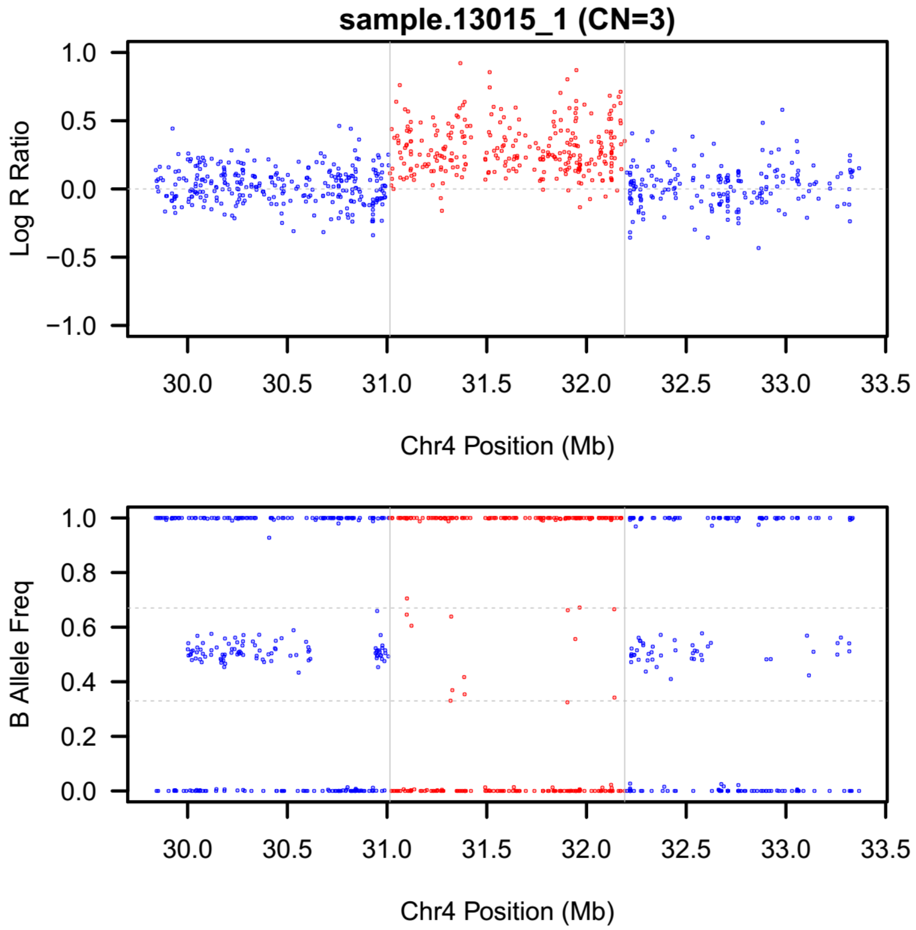

Fig E. (D) A maternally transmitted duplication (blue bar) observed in 1 SA offspring, located downstream of *PCDH7*, a member of the protocadherin gene family with roles in the developing nervous system. CNV boundaries (size) are chr4:31014644-32191478 (~1.2 MB).

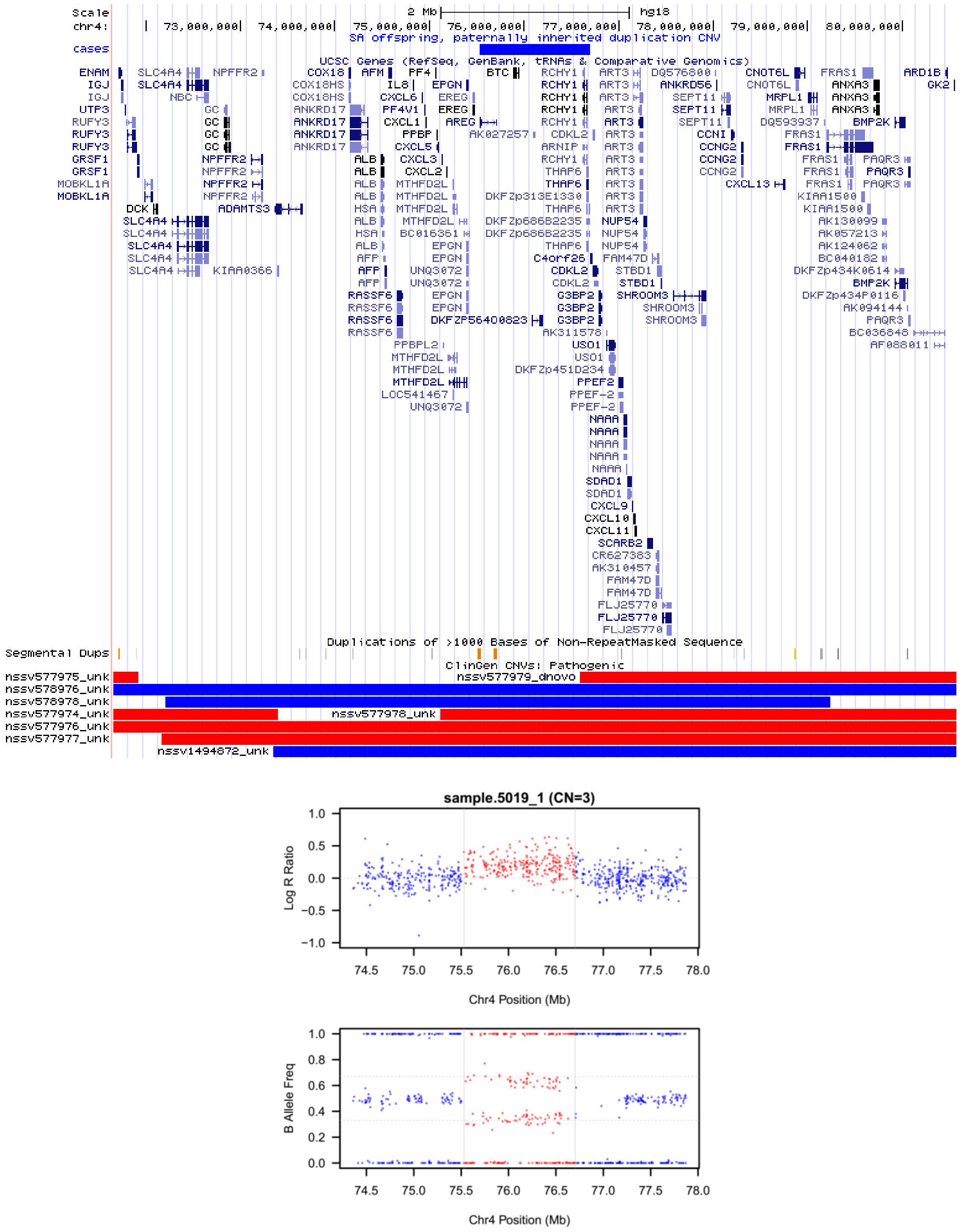

Fig E. (E) A paternally transmitted duplication (blue bar in upper panel) observed in 1 SA offspring, located in a gene-rich region. Three cases of larger (7-43 MB) duplication CNVs spanning this region have been classified as pathogenic by the ISCA consortium (http://dbsearch.clinicalgenome.org/search/; PMID 21844811) with regard to developmental delay (blue bars in lower panel). CNV boundaries (size) are chr4:75528333-76702535 (~1.2 MB).

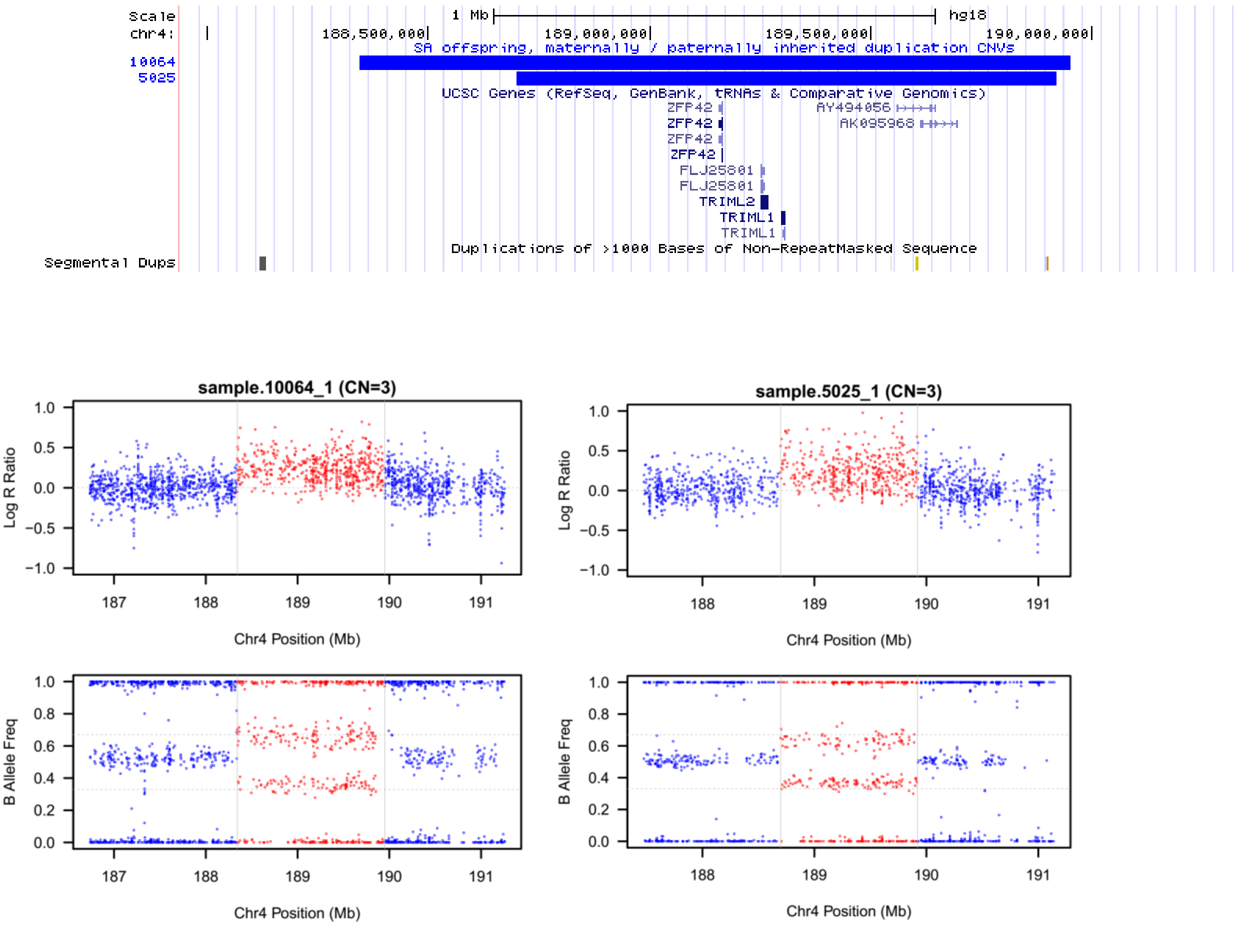

Fig E. (F) Paternally and maternally transmitted duplications (blue bars) observed in 2 SA offspring. *ZFP42* (REX-1) is a zink finger protein involved in transcription, is a well-known embryonic stem cell marker and determines the cell fate in adult stem cells. Several cases of larger (4.5-58 MB) duplication CNVs spanning this region have been classified as pathogenic by the ISCA consortium (http://dbsearch.clinicalgenome.org/search/; PMID 21844811) with regard to intellectual disability, autism or developmental delay. CNV boundaries (size) are chr4:188342786-189950157 (~1.6 MB) and chr4:188698113-189920157 (~1.2MB).

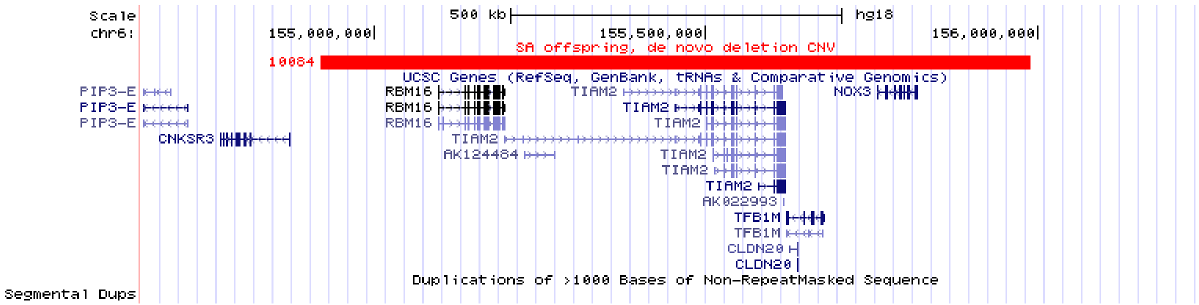

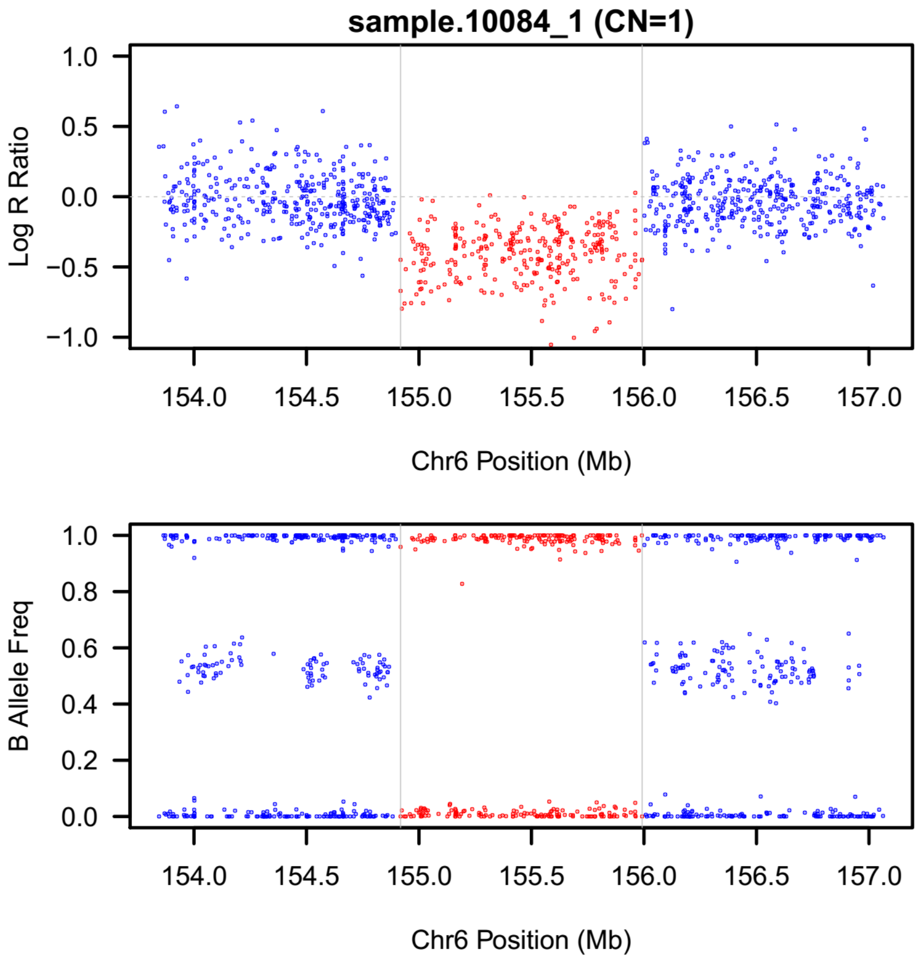

Fig E. (G**)** *De novo* deletion (red bar) observed in 1 SA offspring. *TIAM2* modulates the activity of RHO-like proteins and has roles in neural cell development processes. CNV boundaries (size) are chr6:154918196-155991693 (~1.1 MB).

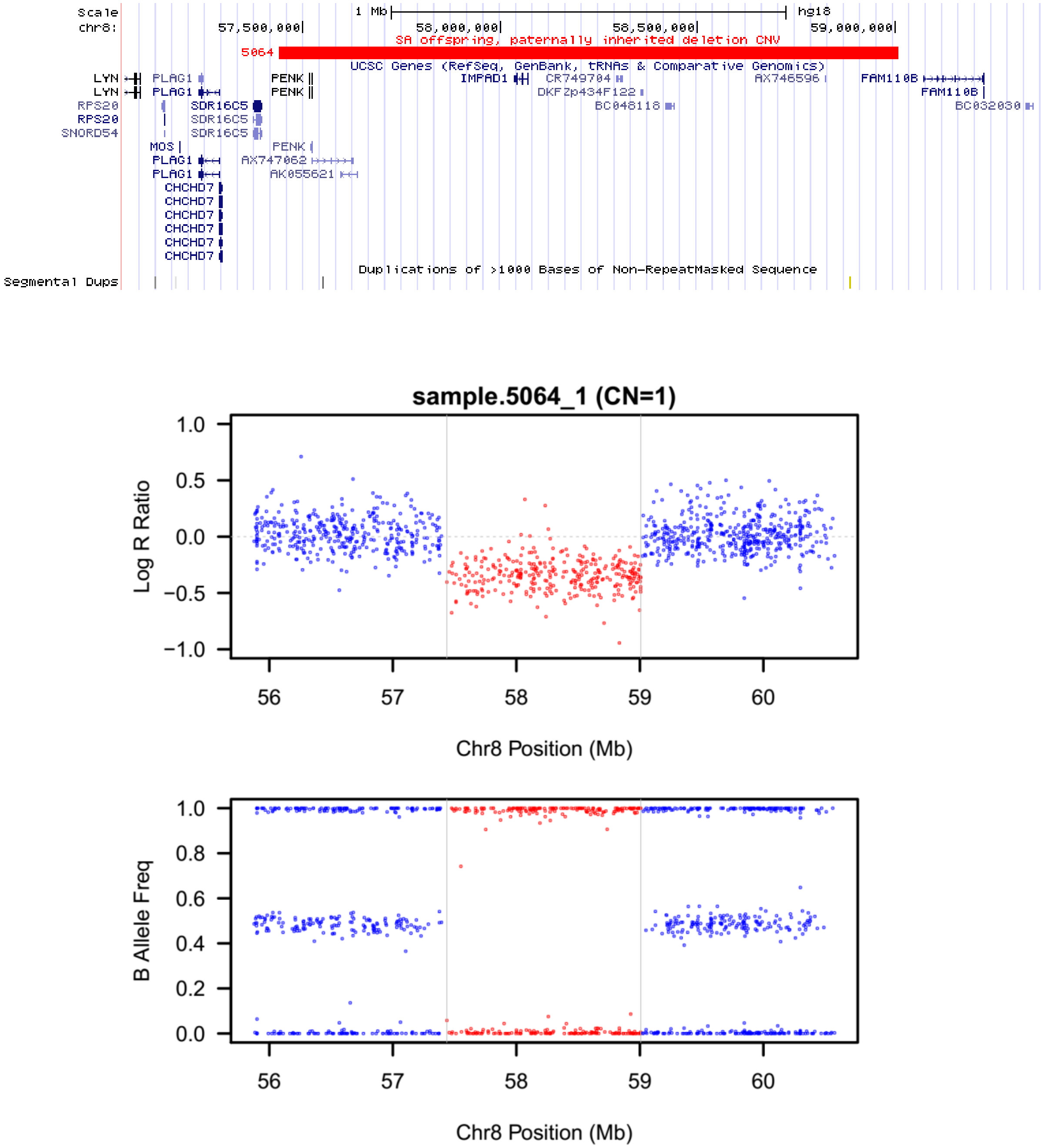

Fig E. (H) A paternally transmitted deletion (red bar) observed in 1 SA offspring. *PENK* (Proenkephalin) encodes endogenous opioids Met-enkephalin and Leu-enkephalin involved in a role in a number of physiologic functions, including pain perception and responses to stress; modulation of glutamate and GABA release. CNV boundaries (size) are chr8:57437447-59006759 (~1.6 MB).

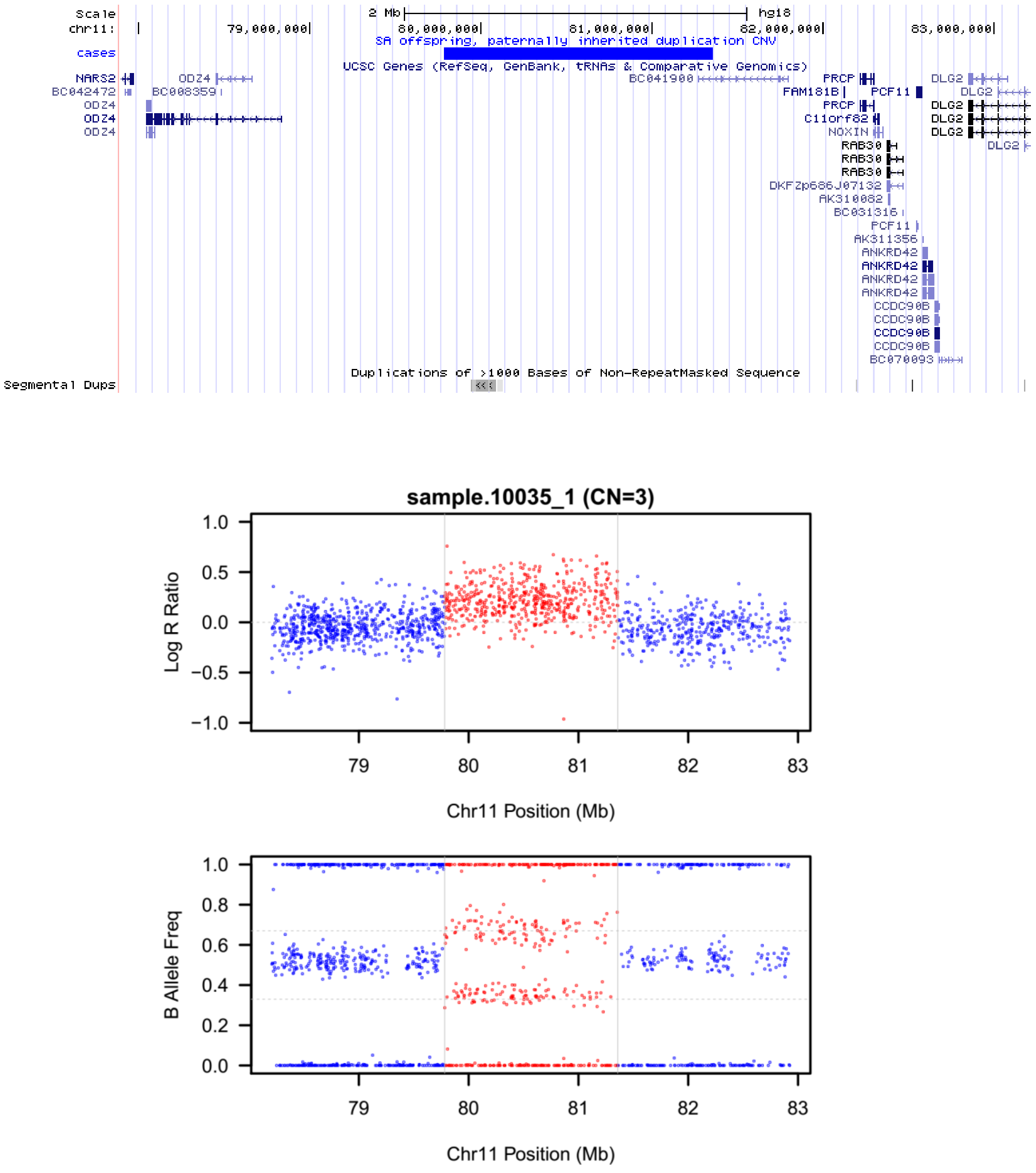

Fig E. (I) A paternally transmitted duplication (blue bar) observed in 1 SA offspring. *ODZ4* (*TENM4*) is involved in neural development and associated with bipolar disorder / schizophrenia in GWAS. *DLG2* has multiple neural roles by regulating e.g. NMDA receptor activity. CNV boundaries (size) are chr11:79782071-81358036 (~1.6 MB).

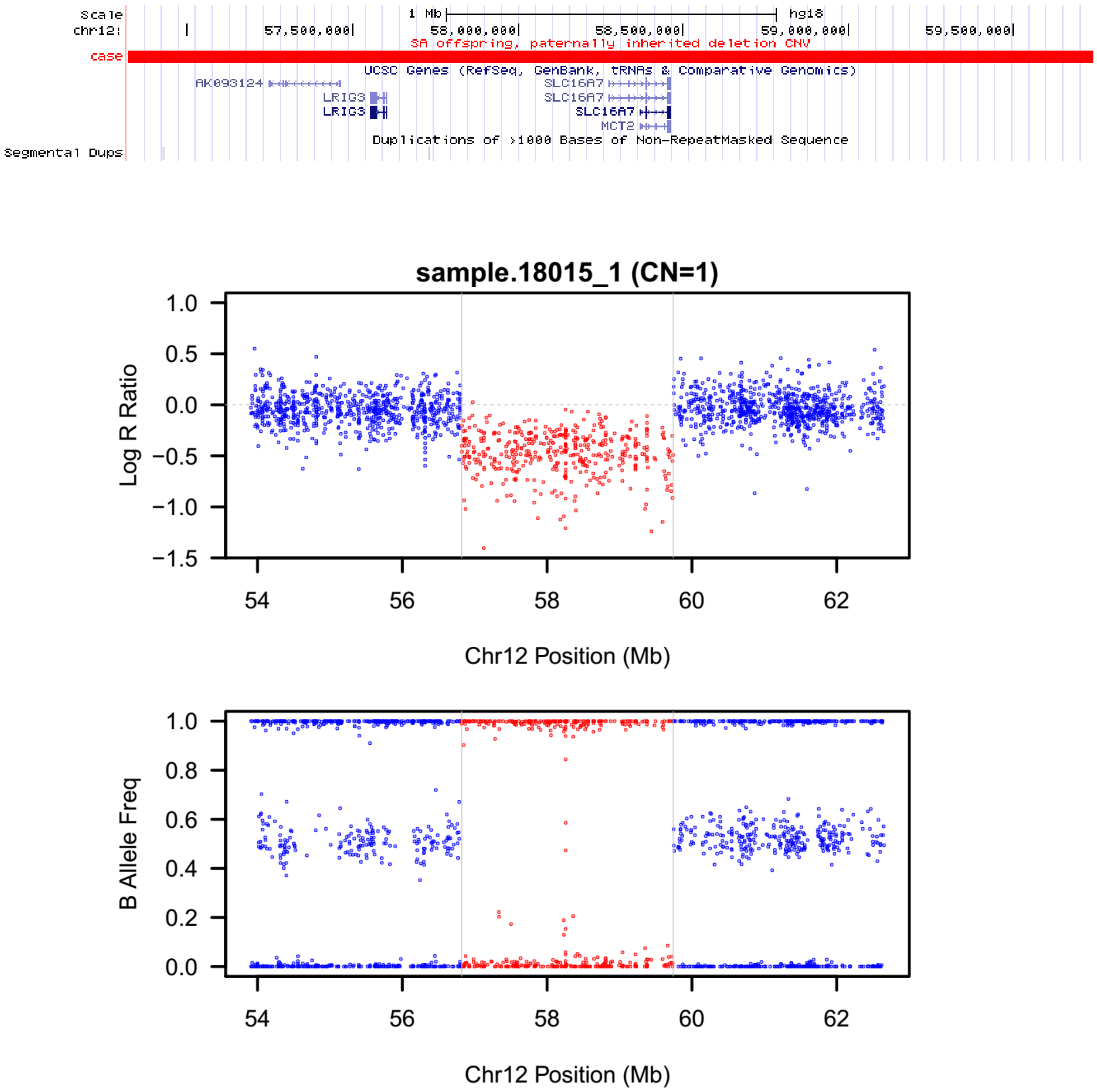

Fig E. (J) A paternally transmitted deletion (red bar) observed in 1 SA offspring. *LRIG3* is a paralog of *LRRTM2*, which play roles in neural system development. CNV boundaries (size) are chr12:56820511-59741054 (~2.9 MB).

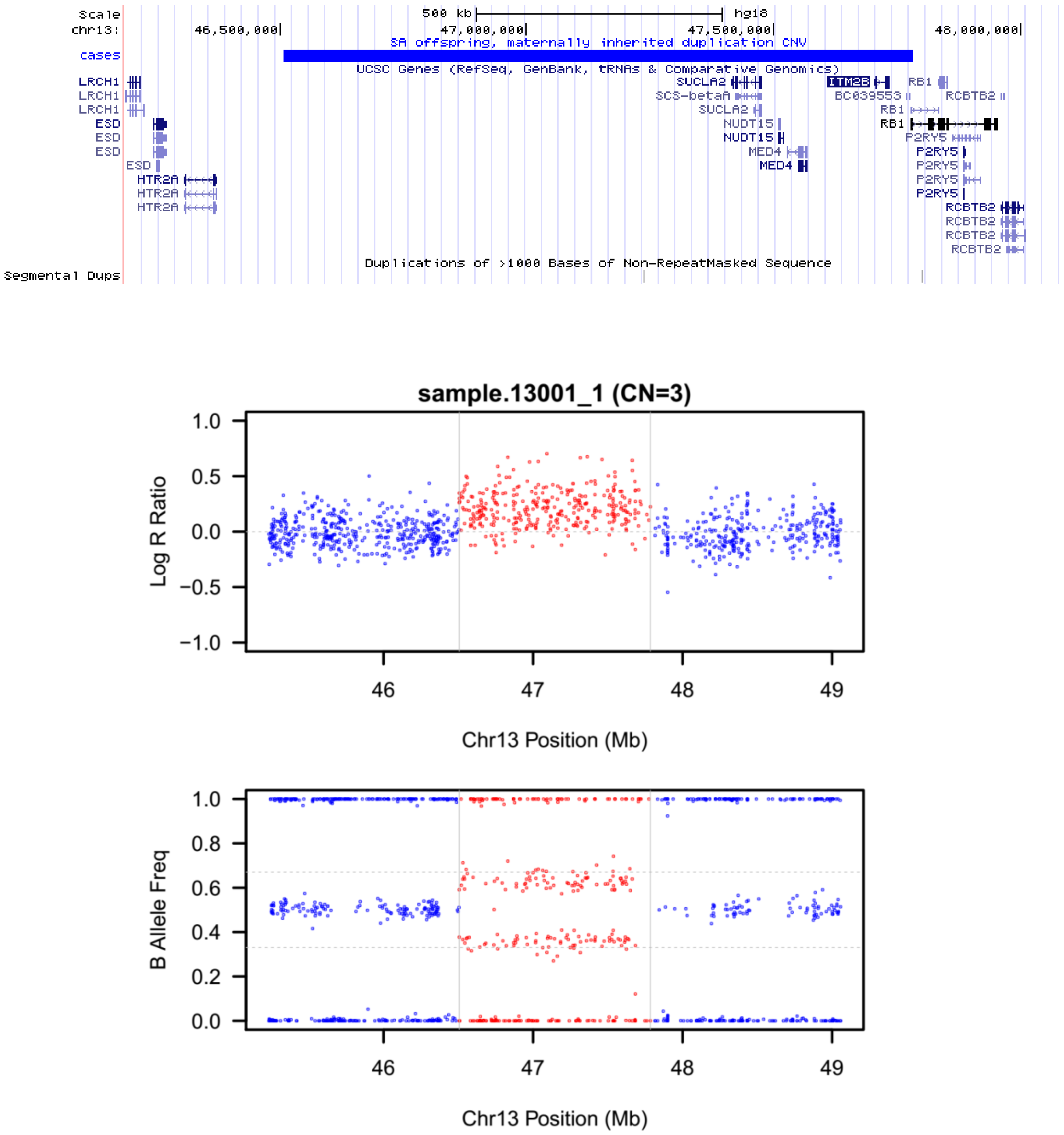

Fig E. (K) A maternally transmitted duplication (blue bar) observed in 1 SA offspring. The duplication is located upstream of SB candidate gene *HTR2A*. *SUCLA2* and *NUTD15* have mainly metabolic functions, while *MED4* and *RB1* are cell-cycle regulators involved in retinoblastoma. CNV boundaries (size) are chr13:46505958-47784283 (~1.3 MB).

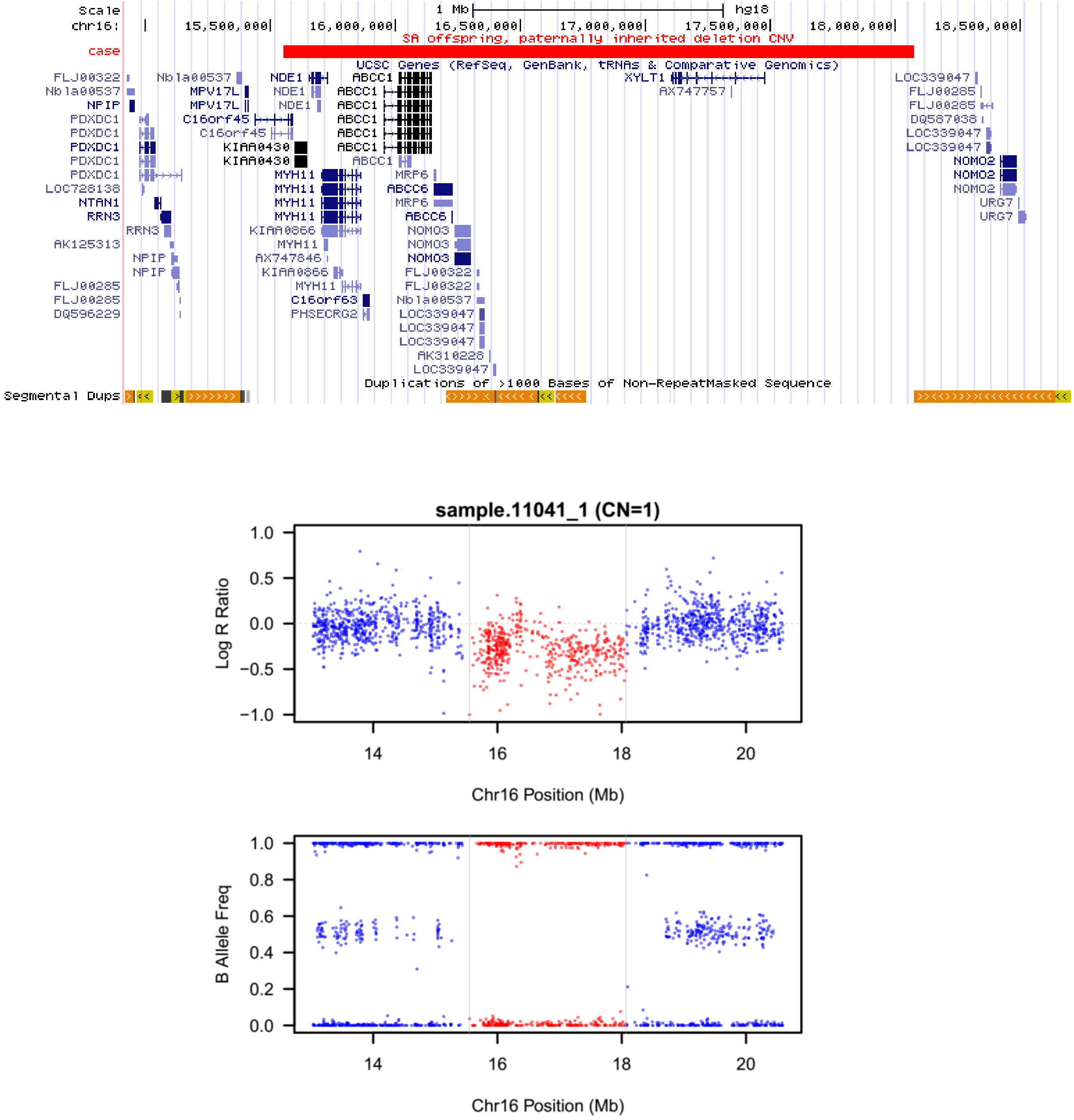

Fig E. (L) A paternally transmitted deletion (red bar) observed in 1 SA offspring. *NDE1* is the NudE Neurodevelopment Protein 1 and mutations therein may cause lissencephaly 4 characterized by brain atrophy and mental retardation. *ABCC1* (MRP1) is an ATP-Binding Cassette transporter involved in e.g. multidrug resistance and neuroprotection (PMID 26187753), and other ABC transporters have been studied as SB candidate genes. CNV boundaries (size) are chr16:15550310-18070334 (~2.5 MB).

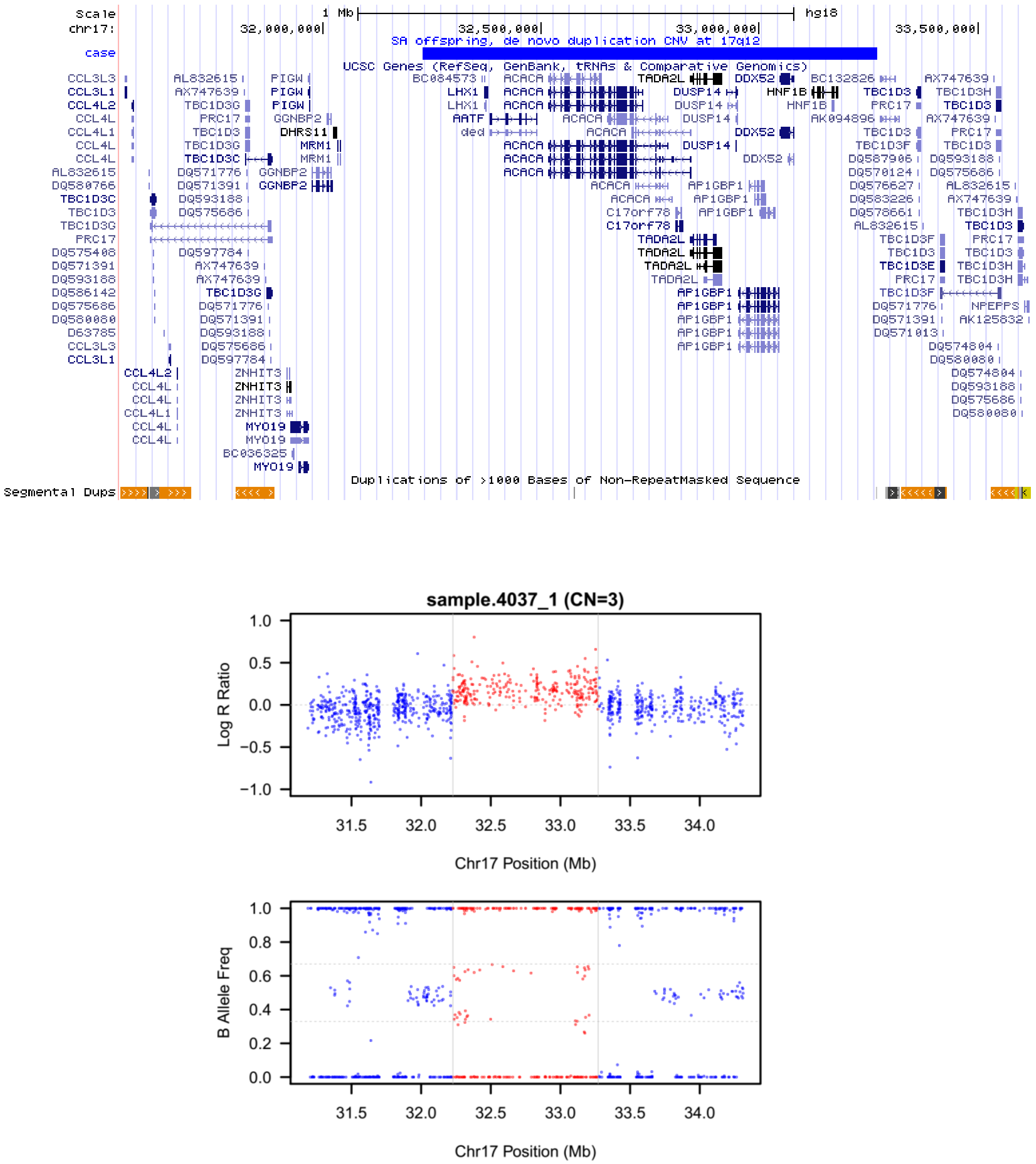

Fig E. (M) Maternally transmitted duplication (blue bar) observed in 1 SA offspring, located in the 17q12 Schizophrenia-associated and developmental 17q12 microdeletion syndrome region. CNV boundaries (size) are chr17:32227936-33270047 (~1 MB).

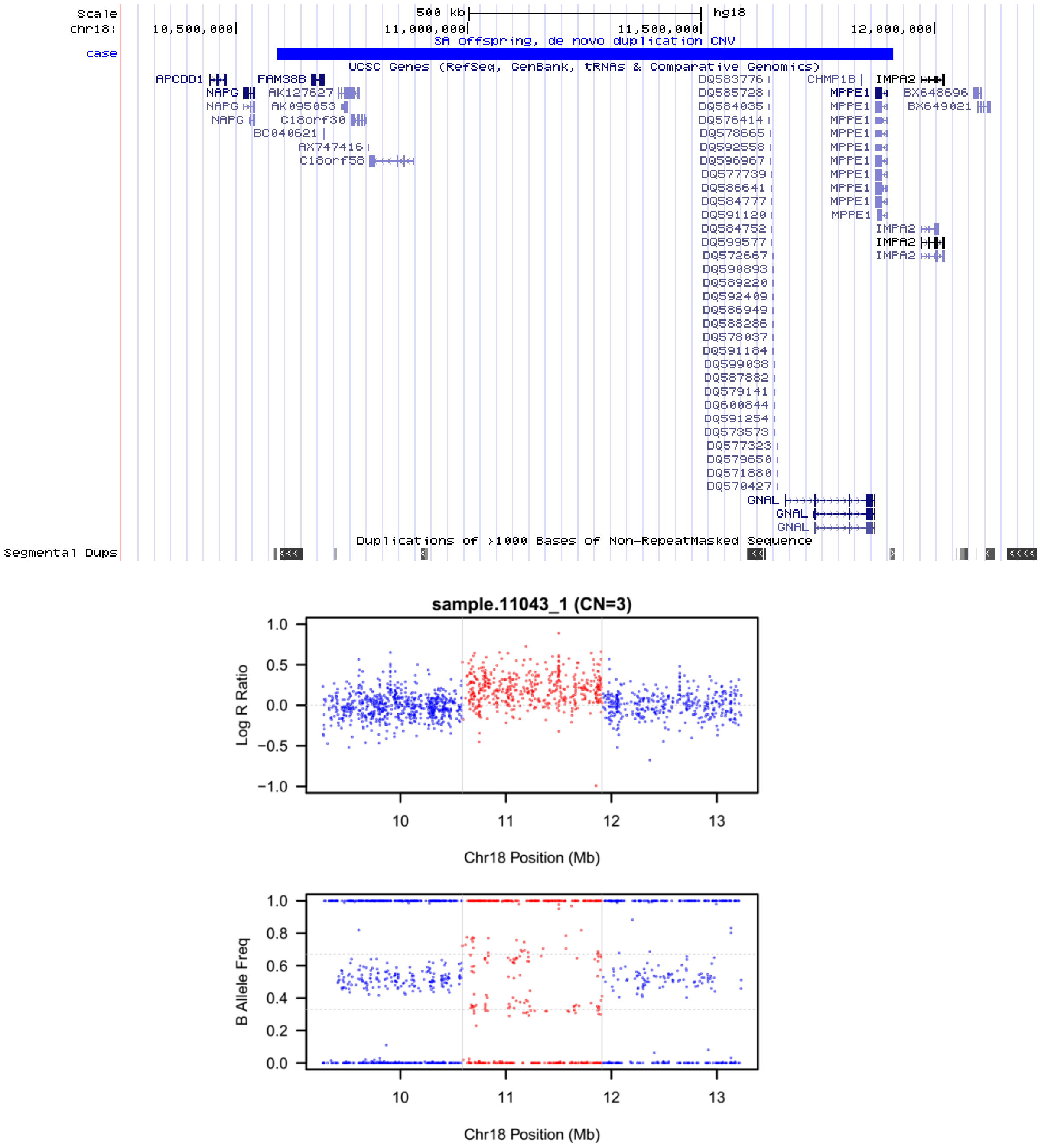

Fig E. (N) *De novo* duplication (blue bar) observed in 1 SA offspring. *FAM32B* (*PIEZO2*) functions as part of mechanically-activated (MA) cation channels in somatosensory neurons and defects may cause type 5 distal arthrogryposis. *GNAL* (*DYT25*) is a brain expressed G protein alpha subunit mediating the signal transduction of several GCPRs and mutations therein may cause adult-onset cranial-cervical dystonia. CNV boundaries (size) are chr18:10587874-11910658 (~1 .3MB).

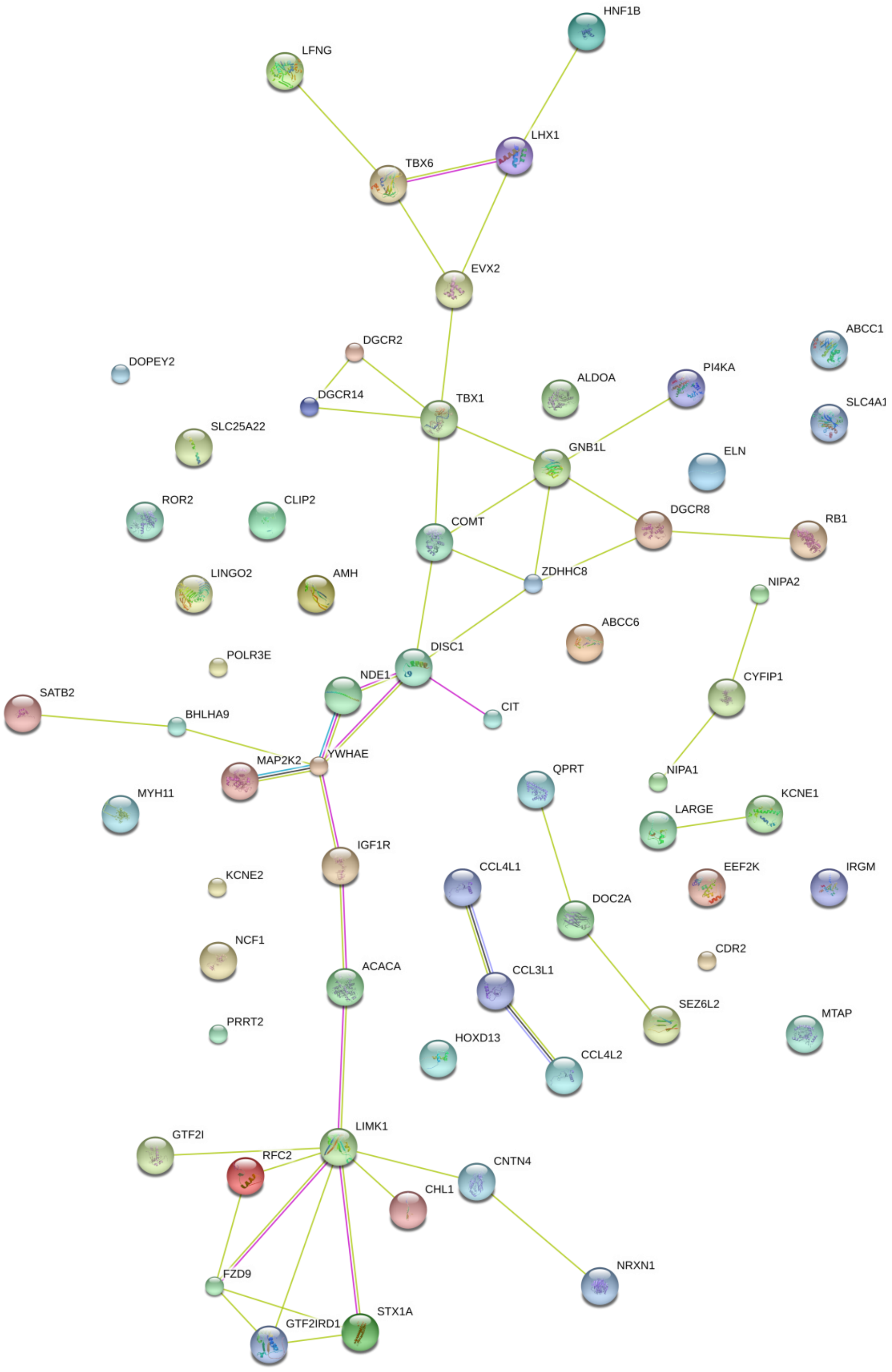

Fig F. A significantly connected PPI network according to the STRING v.10 server [6] for the 65 medically relevant and CNV-affected genes, which were found here in among SA offspring CNVs.

Table A. Significant FBAT-CNV associations mapped to T-cell receptor regions.

Marker ID	Chr	Marker position[1]	Consensus CNV [2]	CNV position[1] (start .. stop)	CNV size (kb)	Type [2]
cnvi0108946	14	22,794,779	CNVR2823	22,315,506..23,005,311	690	Gain+Loss
cnvi0118308	14	22,582,820	CNVR2823	22,315,506..23,005,311	690	Gain+Loss
rs8010032	14	22,609,387	CNVR2823	22,315,506..23,005,311	690	Gain+Loss
rs10143357	14	22,788,610	CNVR2823	22,315,506..23,005,311	690	Gain+Loss
rs17116313	14	22,781,112	CNVR2823	22,315,506..23,005,311	690	Gain+Loss
rs8016619	14	22,737,346	CNVR2823	22,315,506..23,005,311	690	Gain+Loss
rs1860517	7	38,330,810	CNVR9697	38,282,237..38,418,367	136	Gain+Loss

The TCR alpha locus on chromosome 14 (NCBI gene ID 6955) is located between GRCh37/hg19 positions 22,090,057 and 23,021,075, and the TCR gamma locus on chromosome 7 (NCBI gene ID 445347) is located between GRChg19 positions 38,299,243 and 38,357,285; thus both overlap with the observed CNV-associations observed here.

[1] Illumina marker and consensus CNV position coordinates according to the GRCh37/hg19 built.

[2] ID of consensus CNV in the region according to recent meta-analysis [7], as also presented in the database of genomic variants at http:// http://dgv.tcag.ca [8].

Table B. Filtering CNV calls and trios by the various QC procedures to obtain a set of putative rare (<1%) and large (>100kb) CNVs for burden analyses.

	# Calls (# carriers)			
Filter step:	SA offspring	*de novo*	Mothers	Fathers
Trio-based calls, pre-QC	6100 (660)		6723 (660)	6594 (660)
Post-QC and <10 CNVs per subject	3236 (582)	365 (189)	3167 (582)	3192 (582)
Removed centromeric, telomeric, TCR	2335 (550)		2206 (546)	2239 (540)
CNVs *f*<1%	433 (242)		453 (270)	417 (260)
Merge CNV calls for gaps <20%	424 (242)		429 (270)	403 (260)
Remove CNV calls with >50% SegDup overlap	385 (225)	100(53)	401 (260)	371 (244)

Table C. All 385 putative CNVs in 225 SA offspring carriers.

Family-ID	Position	#SNP	Start-SNP	End-SNP	Size (kb)	Type	Source
11026	chr1:3534206-3639665	50	rs4276857	rs9659688	105	Dup	inherited
10031	chr1:12262206-12698438	93	rs4845898	rs3000861	436	Dup	inherited
23010	chr1:17422708-17527994	62	rs2293912	rs1748041	105	Dup	inherited
23042	chr1:86352483-86461066	34	rs12024954	rs1336050	109	Del	deNovo
15024	chr1:107565447-107944435	128	rs6583016	rs12729735	379	Dup	inherited
18029	chr1:165073806-165229302	47	rs7544940	rs1887536	155	Del	deNovo
25014	chr1:173697687-173906328	66	rs859374	rs12403473	209	Dup	inherited
11007	chr1:213302819-213463166	48	rs12038094	rs12080135	160	Del	inherited
6009	chr1:213331109-213493193	47	rs12133857	rs6540890	162	Del	deNovo
16006	chr1:213374400-213486294	40	rs11120504	rs10494996	112	Del	inherited
19012	chr1:229774037-229879757	32	rs7533573	rs6541281	106	Dup	inherited
8018	chr1:233251908-233458977	32	rs34578818	rs4659674	207	Dup	inherited
13056	chr1:244336874-244562256	91	rs4654197	rs6659024	225	Del	inherited
21023	chr2:747786-851841	68	rs12621177	rs4992454	104	Dup	inherited
5069	chr2:27436777-27637305	43	rs6716894	rs4665382	201	Del	deNovo
10060	chr2:31452327-31659330	65	rs2281547	rs9282858	207	Dup	inherited
13022	chr2:32487073-33176091	166	rs176400	rs218211	689	Dup	inherited
23010	chr2:32934163-33038806	33	rs17012350	rs4952324	105	Del	inherited
13049	chr2:34120683-34266891	46	rs2042583	rs11896394	146	Del	inherited
14002	chr2:35356784-35511395	35	rs4550646	rs4280439	155	Del	inherited
10021	chr2:35674481-35941146	34	rs1371413	rs11885738	267	Del	inherited
19023	chr2:35674481-35941146	34	rs1371413	rs11885738	267	Del	inherited
22017	chr2:35674481-35941146	34	rs1371413	rs11885738	267	Del	inherited
1109	chr2:50813187-51095774	67	rs858951	rs12467557	283	Del	deNovo
23030	chr2:51136113-51240242	32	rs6545190	rs13428293	104	Del	inherited
16003	chr2:57201764-57337173	46	rs13019473	rs4271799	135	Del	deNovo
23008	chr2:57226235-57328699	35	rs6709593	rs34129174	102	Del	deNovo
6009	chr2:57226235-57341806	38	rs6709593	rs4619596	116	Del	inherited
4035	chr2:57227769-57341806	37	rs1582644	rs4619596	114	Del	deNovo
23011	chr2:57227769-57328699	34	rs1582644	rs34129174	101	Del	inherited
21006	chr2:68464626-68583496	31	rs17035395	rs11902811	119	Del	inherited
10011	chr2:68467214-68623780	38	rs2070171	rs7560955	157	Del	deNovo
23008	chr2:68474434-68618709	35	rs1542136	rs13404469	144	Del	deNovo
11051	chr2:68474434-68616579	34	rs1542136	rs10172469	142	Del	deNovo
4044	chr2:83278042-83454796	36	rs1430286	rs1991542	177	Del	deNovo
21026	chr2:85243196-85377880	66	rs6547597	rs6737773	135	Dup	inherited
1140	chr2:126574674-126778836	41	rs28715475	rs360263	204	Del	deNovo
1090	chr2:129707339-130200380	119	rs13033217	rs4233595	493	Del	inherited

5065	chr2:130610467-130847995	47	rs4474835	rs3739122	238	Del	inherited
18013	chr2:130665320-130808693	34	rs1811500	rs949727	143	Del	deNovo
23042	chr2:135407661-135545779	31	rs6705067	rs6736412	138	Del	deNovo
51001	chr2:143279164-143646387	72	rs6717886	rs10176686	367	Del	inherited
7001	chr2:161972461-162073448	31	rs2018759	rs7588198	101	Del	inherited
4044	chr2:169987194-170266477	73	rs6731329	rs11685651	279	Del	deNovo
23010	chr2:176643771-176756047	54	rs1000032	rs1446575	112	Del	inherited
22003	chr2:188229479-188490461	31	rs10203300	rs6732052	261	Del	inherited
4048	chr2:198874627-199903114	163	rs13387754	rs1011833	1028	Dup	inherited
22056	chr2:240751883-240878363	55	rs13017098	rs7565801	126	Dup	inherited
22042	chr3:57010-1054024	521	rs1516321	rs7631088	997	Dup	inherited
22060	chr3:1134787-2375967	828	rs13069565	rs6785626	1241	Dup	inherited
5032	chr3:2899986-3102519	164	rs13089241	rs3804800	203	Dup	inherited
10084	chr3:31077039-31260085	54	rs6550039	rs13320098	183	Dup	inherited
13001	chr3:37319184-37498640	55	rs11129756	rs391224	179	Dup	inherited
23015	chr3:55003070-55480430	219	rs1040168	rs11918967	477	Dup	inherited
8063	chr3:55005244-55480430	217	rs9851733	rs11918967	475	Dup	inherited
22032	chr3:73803948-74117031	140	rs4599238	rs6771004	313	Dup	inherited
16006	chr3:99825597-99971701	35	rs1000003	rs3755573	146	Del	inherited
23011	chr3:99825597-99926338	30	rs1000003	rs844159	101	Del	inherited
11028	chr3:117706380-118141555	136	rs3853084	rs3972692	435	Del	inherited
22035	chr3:144152746-144301967	43	rs6769643	rs936198	149	Del	deNovo
18029	chr3:144221793-144342797	38	rs6440128	rs726737	121	Del	deNovo
1104	chr3:146960115-147192004	56	rs16857193	rs6763296	232	Del	inherited
5061	chr3:180445591-180690286	71	rs6799756	rs6807740	245	Dup	inherited
21035	chr4:1030113-1144202	75	cnvi0119515	rs10014245	114	Dup	inherited
27401	chr4:1037330-1137407	65	cnvi0112494	rs11937228	100	Dup	deNovo
7101	chr4:1370848-1492404	67	rs4974559	cnvi0088904	122	Dup	inherited
5069	chr4:7827957-7930340	52	rs4689859	rs28622214	102	Dup	deNovo
10053	chr4:12215589-12345813	38	rs11722218	rs9884719	130	Del	deNovo
16006	chr4:12215589-12350411	39	rs11722218	rs10022602	135	Del	inherited
1057	chr4:12234478-12345813	33	rs13149659	rs9884719	111	Del	deNovo
1002	chr4:12236260-12345813	32	rs1545817	rs9884719	110	Del	inherited
5007	chr4:12240925-12366540	33	rs10032940	rs7692324	126	Del	inherited
11007	chr4:12240925-12345813	31	rs10032940	rs9884719	105	Del	inherited
10028	chr4:14412892-14543205	58	rs4298137	rs11722368	130	Del	deNovo
16006	chr4:14438686-14571594	52	rs11930642	rs6449093	133	Del	inherited
10053	chr4:14441104-14581659	52	rs4569748	rs1494948	141	Del	deNovo
16010	chr4:14441104-14581659	52	rs4569748	rs1494948	141	Del	inherited
21023	chr4:14446386-14615855	72	rs10939594	cnvi0079864	169	Del	deNovo
12001	chr4:14480132-14581659	36	rs28597787	rs1494948	102	Del	deNovo

11007	chr4:14494473-14599987	35	rs17440311	rs13117049	106	Del	inherited
13015	chr4:31014644-32191478	284	rs7689511	rs11941142	1177	Dup	inherited
25030	chr4:34416670-34645722	43	rs13130083	rs2370152	229	Del	deNovo
5047	chr4:39581810-39698906	30	rs6531748	rs7686844	117	Del	inherited
25006	chr4:65925144-66181611	83	rs10012882	rs11735820	256	Dup	inherited
4044	chr4:71473943-71849025	51	rs1490470	rs2278134	375	Del	deNovo
16006	chr4:71476784-71759750	34	rs17149035	rs16845359	283	Del	inherited
6009	chr4:71481682-71740586	32	rs17733915	rs7697213	259	Del	inherited
10053	chr4:71487617-71759750	32	rs28661403	rs16845359	272	Del	deNovo
4035	chr4:71487617-71765873	33	rs28661403	rs16845371	278	Del	inherited
1054	chr4:73976543-74277270	47	rs9291180	rs10028486	301	Dup	inherited
5019	chr4:75528333-76702535	271	rs1797605	rs11099642	1174	Dup	inherited
16003	chr4:75543154-75756795	35	rs1691274	rs12647729	214	Del	deNovo
10020	chr4:98049258-98288034	44	rs17026062	rs7693495	239	Del	inherited
8022	chr4:115785108-116619098	249	rs13152031	rs6533913	834	Dup	inherited
10060	chr4:120449894-120621283	37	rs4834769	rs10518329	171	Del	deNovo
18034	chr4:132165825-132573737	54	rs6822842	rs9993647	408	Dup	inherited
1126	chr4:132165825-132573737	54	rs6822842	rs9993647	408	Dup	inherited
19014	chr4:132165825-132573737	54	rs6822842	rs9993647	408	Dup	inherited
25007	chr4:132338035-132793488	74	rs17447684	rs1500777	455	Del	inherited
1116	chr4:147202690-147584620	103	rs1426888	rs13105153	382	Dup	inherited
11007	chr4:168265710-168375955	33	rs1579404	rs9312517	110	Del	inherited
16006	chr4:168316692-168427667	35	rs17703374	rs13117708	111	Del	deNovo
10064	chr4:188342786-189950157	630	rs931370	rs1461780	1607	Dup	inherited
5025	chr4:188698113-189920157	528	rs10032493	rs13146779	1222	Dup	inherited
5024	chr5:784584-937439	33	rs421718	rs10072022	153	Dup	deNovo
24006	chr5:784584-937439	33	rs421718	rs10072022	153	Dup	inherited
10075	chr5:12802695-13004160	48	rs6862501	cnvi0145079	201	Del	inherited
15010	chr5:18885079-18990473	43	rs12656706	rs4309976	105	Del	inherited
18015	chr5:25051322-25313474	40	rs6869061	rs16894284	262	Dup	inherited
18003	chr5:28360404-28609696	57	rs1006069	rs9292273	249	Dup	inherited
8019	chr5:33990268-34159690	57	rs2287949	rs297622	169	Dup	inherited
23010	chr5:43540505-43652459	34	rs4074777	rs7737947	112	Del	deNovo
1079	chr5:54860351-55055155	41	cnvi0160567	rs35350870	195	Del	inherited
5050	chr5:54860351-55041165	36	cnvi0160567	rs11741159	181	Del	inherited
4044	chr5:60569272-60700113	33	rs7446842	rs7700289	131	Del	deNovo
1079	chr5:65221177-65324547	37	rs2113030	rs27132	103	Del	inherited
21003	chr5:81108735-81209930	33	rs9791117	rs2897524	101	Dup	inherited
25030	chr5:121217395-121348171	33	rs1560550	rs10077959	131	Del	deNovo
22035	chr5:150082437-150312674	83	rs17111302	rs11167526	230	Dup	deNovo
18013	chr5:150156524-150260690	58	rs9885376	rs10035221	104	Dup	inherited

1058	chr5:178566572-179443248	386	rs457957	rs33916112	877	Del	deNovo
18006	chr6:4199051-4417586	56	cnvi0156933	rs7746329	219	Del	inherited
23003	chr6:27754471-27887485	112	rs13210634	rs200486	133	Dup	deNovo
11013	chr6:67872242-68242949	42	rs9454034	rs10498856	371	Del	inherited
5051	chr6:78053609-78185841	41	rs9448054	rs9359267	132	Dup	inherited
1028	chr6:78066651-78185841	40	cnvi0130877	rs9359267	119	Dup	inherited
10035	chr6:95132646-95244994	33	rs9452659	rs9445285	112	Del	inherited
10084	chr6:154918196-155991693	439	rs17085526	rs9480176	1073	Del	deNovo
18025	chr6:167603059-167750675	110	rs4710230	rs13198757	148	Dup	inherited
1117	chr6:169346057-169654484	109	rs7756742	rs2997887	308	Dup	inherited
27401	chr7:1309351-1503243	47	rs13230410	rs3735660	194	Dup	deNovo
21004	chr7:1608435-1717323	37	rs11767554	rs4720940	109	Dup	deNovo
1047	chr7:1651536-1759330	31	rs7785046	rs4719278	108	Dup	inherited
1099	chr7:2527303-2659332	55	rs4632959	rs6461505	132	Del	deNovo
4048	chr7:4259582-4369056	56	rs12531029	rs6951614	109	Dup	inherited
4054	chr7:7307919-7480760	73	rs7777201	rs6952195	173	Dup	inherited
1138	chr7:8923566-9160323	59	rs10266202	rs13246476	237	Del	inherited
22034	chr7:8928558-9110765	52	rs17157252	rs12538490	182	Del	inherited
19014	chr7:12509645-12622940	51	rs13246109	rs849798	113	Dup	inherited
1079	chr7:23603941-23723687	32	rs227937	rs6945306	120	Del	inherited
13032	chr7:29175877-29503232	181	rs245908	rs10085463	327	Dup	inherited
5064	chr7:29175877-29503232	181	rs245908	rs10085463	327	Dup	inherited
1038	chr7:56512872-56808838	62	rs7806502	rs36096266	296	Dup	inherited
10065	chr7:64315501-64579115	77	cnvi0160144	cnvi0070617	264	Del	deNovo
12003	chr7:64315501-64602751	78	cnvi0160144	cnvi0153876	287	Del	inherited
15024	chr7:64315501-64602751	78	cnvi0160144	cnvi0153876	287	Del	inherited
1076	chr7:64315501-64602751	78	cnvi0160144	cnvi0153876	287	Del	inherited
10087	chr7:64365027-64616251	52	rs17139613	cnvi0159098	251	Dup	inherited
8058	chr7:64365027-64602751	47	rs17139613	cnvi0153876	238	Del	inherited
16010	chr7:67411180-67550019	32	rs3113166	rs12540706	139	Del	inherited
84018	chr7:72488114-73875272	356	rs1178947	cnvi0143742	1387	Dup	deNovo
13051	chr7:100755083-100922118	45	rs7799285	rs12537163	167	Dup	inherited
13038	chr7:100755083-100914175	44	rs7799285	rs13232646	159	Dup	inherited
18034	chr7:100755083-100914175	44	rs7799285	rs13232646	159	Dup	inherited
21023	chr7:100878190-100981144	49	rs869127	rs17135626	103	Dup	deNovo
22030	chr7:102396457-102588587	35	rs11514917	cnvi0076001	192	Del	inherited
11007	chr7:108863420-108978071	31	rs2091546	rs1357687	115	Del	inherited
4042	chr7:123176329-123606391	73	rs4731119	rs11560338	430	Dup	inherited
13038	chr7:124774129-125577911	98	rs17148210	rs17656707	804	Del	inherited
6009	chr7:141375091-141494251	37	rs12540186	rs6954522	119	Del	deNovo
16006	chr7:141376097-141494251	35	rs6956284	rs6954522	118	Del	deNovo

18029	chr7:148397324-148571806	47	rs6961306	rs10241873	174	Del	deNovo
23010	chr7:148397324-148557538	41	rs6961306	rs1724329	160	Del	deNovo
13015	chr8:2334274-2570605	121	rs4875971	rs6558680	236	Dup	inherited
1102	chr8:2399687-2663875	135	rs17070319	rs7813089	264	Dup	inherited
25016	chr8:4271081-4424974	49	rs17414486	rs2725065	154	Del	inherited
10099	chr8:4274542-4403280	43	rs7015706	rs17344322	129	Del	inherited
13010	chr8:5581218-6250491	343	rs859812	rs1057187	669	Del	inherited
4028	chr8:8357335-8486098	37	rs2921054	rs12541355	129	Del	inherited
5035	chr8:16963457-17080632	48	rs17624806	rs2517082	117	Dup	inherited
10028	chr8:33253317-33355213	36	rs7833088	rs7837460	102	Del	deNovo
6009	chr8:33253317-33355213	36	rs7833088	rs7837460	102	Del	inherited
22017	chr8:33263472-33388156	42	rs4427132	rs12541652	125	Del	inherited
5064	chr8:57437447-59006759	328	rs4599805	rs12707981	1569	Del	inherited
10022	chr8:69981530-70129490	48	cnvi0001841	rs2162353	148	Dup	inherited
25030	chr8:89407271-89654164	34	rs7835845	rs7838490	247	Del	deNovo
18029	chr8:96462846-96618570	35	rs3103764	rs1392793	156	Del	inherited
22025	chr8:103360767-103617738	40	rs2512408	rs1347322	257	Dup	inherited
8061	chr9:1363944-1474527	74	rs646785	rs4741416	111	Dup	inherited
23027	chr9:2597430-3000275	169	rs10812332	rs2889463	403	Dup	inherited
1048	chr9:2606042-2710701	67	rs11790563	rs16909365	105	Dup	inherited
22054	chr9:4165223-4297572	114	rs7044458	rs10814921	132	Dup	inherited
8041	chr9:6024076-6332855	71	rs899381	rs2381438	309	Del	inherited
18013	chr9:6608004-6712212	53	rs10120677	rs2990659	104	Del	deNovo
8043	chr9:6685397-6900916	75	rs1094038	rs12235748	216	Dup	inherited
1095	chr9:11957101-12360395	94	cnvi0159513	rs10809762	403	Del	inherited
5012	chr9:21718683-21847303	49	rs6475564	rs7047899	129	Dup	inherited
13049	chr9:28603702-28835945	33	rs12347952	rs12004937	232	Del	inherited
12001	chr9:28652170-28837621	40	cnvi0123992	cnvi0064238	185	Del	inherited
8014	chr9:30450743-31055183	73	rs10813257	rs7866107	604	Del	inherited
1122	chr9:65597304-65771759	38	cnvi0067121	cnvi0121544	174	Del	inherited
1129	chr9:93269250-93470947	185	rs12345170	rs9409640	202	Dup	inherited
10021	chr9:103914805-104049649	36	rs1375707	rs10989911	135	Del	inherited
16010	chr9:104746290-104862217	49	rs2067711	rs1099151	116	Del	inherited
10028	chr9:112414686-112549599	57	rs4302915	rs2846448	135	Del	deNovo
12001	chr9:116030816-116143794	80	rs4979357	rs2250242	113	Dup	inherited
19022	chr9:117162449-117927770	228	rs4979521	rs204505	765	Dup	inherited
23010	chr9:136587114-136689686	69	rs11103276	cnvi0104145	103	Dup	deNovo
8041	chr9:137194583-137424099	161	rs10124337	rs4841972	230	Dup	inherited
21013	chr9:137286630-137449867	107	rs11792631	rs35532724	163	Dup	inherited
1079	chr9:137288987-137449867	105	rs7858253	rs35532724	161	Dup	inherited
21023	chr9:137312610-137424099	81	rs7026842	rs4841972	111	Dup	deNovo

18013	chr10:5597678-5705151	41	rs4529812	rs6602300	107	Del	deNovo
16010	chr10:8340796-8460024	58	rs7092384	rs12778305	119	Del	inherited
11013	chr10:16891940-17121159	143	rs11254210	rs11254339	229	Dup	inherited
1077	chr10:19456340-19871538	65	rs12761196	rs12359487	415	Del	inherited
4061	chr10:41932381-42167723	31	cnvi0141177	cnvi0140355	235	Dup	deNovo
1100	chr10:41932381-42167723	31	cnvi0141177	cnvi0140355	235	Del	deNovo
10021	chr10:44513066-44679489	41	rs10900094	rs6593445	166	Dup	inherited
13058	chr10:44529447-44679489	38	rs12772796	rs6593445	150	Dup	inherited
23015	chr10:44529447-44679489	38	rs12772796	rs6593445	150	Dup	inherited
1094	chr10:44545156-44679489	36	rs4948912	rs6593445	134	Dup	inherited
5019	chr10:51633418-51833997	92	cnvi0139901	rs884880	201	Dup	inherited
21023	chr10:80210603-80316545	45	rs16936416	rs2789979	106	Dup	deNovo
16009	chr10:81633431-81872350	92	rs2244701	rs3851050	239	Dup	inherited
27401	chr10:82193486-82297097	47	rs4934167	rs1870136	104	Del	deNovo
27401	chr10:134274251-134414821	48	cnvi0160481	rs12776770	141	Dup	deNovo
5071	chr11:706765-836070	77	rs12222447	rs11246331	129	Dup	inherited
8009	chr11:7754291-7890189	44	rs7940558	rs12291194	136	Del	inherited
22044	chr11:21779778-22113033	38	rs1945339	rs11827461	333	Del	inherited
1039	chr11:26950951-27192892	81	rs1900060	rs4923434	242	Dup	inherited
23042	chr11:32811619-33012262	37	rs12800054	rs6484620	201	Del	deNovo
1002	chr11:34609700-34734927	50	rs286914	rs7110293	125	Dup	inherited
7024	chr11:34631051-34734927	32	rs286896	rs7110293	104	Dup	inherited
22050	chr11:38429958-39000122	50	rs10501199	rs6485050	570	Del	inherited
8048	chr11:48347783-48867768	69	rs17800980	cnvi0139479	520	Del	inherited
10035	chr11:79782071-81358036	534	rs1479300	rs1955011	1576	Dup	inherited
10028	chr11:87305775-87432863	49	rs7109594	rs1386329	127	Del	deNovo
16006	chr11:87319873-87452484	51	rs7108223	rs10831352	133	Del	inherited
5007	chr11:87322513-87431135	42	rs10898804	rs988975	109	Del	inherited
1033	chr11:93109540-93709664	183	rs34591613	rs481597	600	Dup	inherited
25030	chr11:102658129-102927145	91	rs12574626	rs1481996	269	Del	deNovo
4035	chr11:113915872-114034798	40	rs578014	rs4938118	119	Del	deNovo
18028	chr11:133853184-134212629	256	rs10791373	rs2846173	359	Dup	inherited
5012	chr11:133873030-134107173	137	rs4268526	rs11223972	234	Dup	inherited
4044	chr12:38714828-38904364	59	rs1994090	rs1388597	190	Del	deNovo
4035	chr12:38729243-38896150	53	rs10784356	rs2638272	167	Del	inherited
18015	chr12:56820511-59741054	440	rs1109125	rs1472835	2921	Del	inherited
11034	chr12:84406048-84800478	59	rs10779162	rs11117072	394	Dup	inherited
27401	chr12:84547083-84917644	72	rs10863024	rs7300073	371	Dup	inherited
10024	chr12:108036678-108147964	43	rs34261	rs4766584	111	Dup	inherited
10059	chr12:108036678-108147964	43	rs34261	rs4766584	111	Dup	inherited
21035	chr12:112745620-113036905	127	rs2059598	rs4767219	291	Dup	inherited

25006	chr12:112749210-113036905	126	rs11066776	rs4767219	288	Dup	inherited
4061	chr12:112759696-112894524	51	rs7137339	rs999445	135	Dup	inherited
18013	chr12:112977336-113223344	108	rs1076531	rs1896003	246	Dup	deNovo
4044	chr12:118727882-118912851	44	rs10774515	rs6490278	185	Del	deNovo
16010	chr12:119756117-119888107	43	rs11065286	rs7953249	132	Del	inherited
22035	chr12:126152260-126618589	182	rs7965456	rs10444467	466	Dup	inherited
22024	chr12:126891452-127032431	47	rs10847465	rs1471957	141	Del	inherited
11051	chr12:127177345-127551441	218	rs9300283	rs11059722	374	Dup	inherited
22050	chr12:127934640-128066583	62	rs1798911	rs543533	132	Dup	inherited
5040	chr12:127947706-128066583	60	rs11060001	rs543533	119	Dup	inherited
4034	chr12:128372765-129046139	336	rs2128280	rs1499931	673	Dup	inherited
27401	chr12:130979672-131138206	31	rs12307678	rs6598199	159	Dup	deNovo
7014	chr12:131578893-131685117	60	rs12316422	cnvi0126384	106	Dup	inherited
1079	chr13:20473001-20580068	38	rs9316060	rs4770114	107	Del	inherited
21012	chr13:22248509-22394997	49	rs9580437	rs9506969	146	Dup	inherited
18001	chr13:23122142-23377173	61	rs9553023	rs12859875	255	Del	inherited
16012	chr13:42444618-42625076	64	rs9533322	rs2589312	180	Dup	inherited
13001	chr13:46505958-47784283	334	rs11616518	rs2854342	1278	Dup	inherited
23039	chr13:47227772-47587432	93	rs3862745	rs1410117	360	Dup	inherited
18003	chr13:58561359-59277448	238	rs2321997	rs341530	716	Dup	inherited
6009	chr13:61397472-61564115	38	rs7322457	rs7319860	167	Del	inherited
4044	chr13:61409439-61594422	44	rs2183953	rs6562240	185	Del	deNovo
16010	chr13:61409439-61564115	36	rs2183953	rs7319860	155	Del	inherited
11007	chr13:61409439-61564115	36	rs2183953	rs7319860	155	Del	inherited
16006	chr13:61409439-61555336	34	rs2183953	cnvi0049938	146	Del	inherited
5007	chr13:61409439-61555336	34	rs2183953	cnvi0049938	146	Del	inherited
5065	chr13:61797614-61902984	30	rs429397	rs9592187	105	Del	inherited
18005	chr13:71012389-71217866	30	rs17088321	rs7322922	205	Dup	deNovo
23042	chr13:74962410-75097667	49	rs9573570	rs9530453	135	Del	deNovo
13018	chr13:78012972-78118900	33	rs9593339	rs12585725	106	Del	deNovo
22008	chr13:85382404-85912495	99	rs4343164	rs1502067	530	Del	inherited
25030	chr13:89044951-89251796	56	cnvi0158318	rs9301619	207	Dup	inherited
11019	chr13:89059237-89251796	52	rs1327338	rs9301619	193	Dup	inherited
8009	chr14:20233526-20356515	76	rs17242783	rs10872856	123	Dup	inherited
23008	chr14:22224585-22334110	47	rs35720937	rs8008860	110	Dup	inherited
8055	chr14:25187715-25488077	123	rs7159800	rs10145810	300	Dup	inherited
10068	chr14:42906276-43316238	65	rs1389565	rs11157365	410	Dup	inherited
4038	chr14:42906276-43316238	65	rs1389565	rs11157365	410	Dup	inherited
15014	chr14:44674154-44868025	35	rs3825625	rs10151310	194	Del	inherited
10089	chr14:59421671-59640982	37	rs12881072	rs219310	219	Del	deNovo
13025	chr14:82323305-82872581	136	rs2039476	rs162666	549	Del	inherited

7008	chr14:86091981-86293147	36	rs1289241	rs1891548	201	Del	inherited
10057	chr15:20310802-20654346	79	rs11630745	cnvi0136361	344	Del	inherited
11043	chr15:20310802-20638370	70	rs11630745	rs10152278	328	Dup	inherited
22026	chr15:20384417-20638370	49	cnvi0054195	rs10152278	254	Dup	inherited
25028	chr15:20384417-20638370	49	cnvi0054195	rs10152278	254	Dup	inherited
8034	chr15:20384417-20638370	49	cnvi0054195	rs10152278	254	Dup	inherited
1078	chr15:32481609-32640436	44	cnvi0016492	cnvi0075612	159	Dup	inherited
11032	chr15:32492778-32640436	41	cnvi0075586	cnvi0075612	148	Del	deNovo
21031	chr15:32492778-32640436	41	cnvi0075586	cnvi0075612	148	Del	inherited
1124	chr15:34147943-34263957	33	rs789897	rs1369597	116	Del	inherited
10061	chr15:52691187-52907539	62	rs12592900	rs2681987	216	Dup	inherited
7016	chr15:55969746-56095942	35	rs1866964	rs11858606	126	Del	inherited
21023	chr15:64491503-64629063	32	rs2053005	rs7164579	138	Dup	deNovo
18013	chr15:74963213-75111935	35	rs3812908	rs4078354	149	Del	inherited
23053	chr15:85135364-85268654	34	rs16977988	rs16978088	133	Del	inherited
5020	chr15:93071471-93530762	148	rs7173947	rs290644	459	Dup	inherited
5020	chr15:97271086-97743963	241	rs2684811	rs2289558	473	Dup	inherited
4034	chr16:2131735-2243497	48	rs258281	rs10721	112	Dup	deNovo
8058	chr16:5106601-5271839	33	cnvi0142724	rs7188721	165	Del	inherited
13060	chr16:5500905-5638007	90	rs11639680	rs2191098	137	Dup	inherited
25013	chr16:9019717-9155479	31	rs4985019	rs2965915	136	Dup	inherited
4035	chr16:10320373-10490125	32	rs10153114	rs9937061	170	Del	inherited
8060	chr16:15400547-16198600	544	rs153001	rs8062992	798	Dup	inherited
11041	chr16:15550310-18070334	1091	rs4781673	rs8061491	2520	Del	inherited
13032	chr16:15775065-16162267	377	rs11641649	rs169844	387	Dup	inherited
1121	chr16:18956879-19599084	243	rs8043993	rs1548445	642	Dup	inherited
8040	chr16:21856623-22298757	46	rs2945466	rs12444131	442	Dup	deNovo
10059	chr16:21856623-22328822	47	rs2945466	rs11864229	472	Del	inherited
5053	chr16:29496654-30095177	154	cnvi0140125	rs12373078	599	Dup	deNovo
10011	chr16:61726790-61976280	44	rs10221015	rs1012248	249	Del	inherited
10091	chr16:81046497-81168365	76	rs4782642	rs9938883	122	Dup	inherited
10091	chr16:81283985-81400668	103	rs7195110	rs9319574	117	Dup	inherited
23010	chr16:86820113-86936870	31	rs11864961	rs7198446	117	Dup	deNovo
4026	chr16:87020736-87128478	56	rs7201175	cnvi0091091	108	Dup	inherited
7101	chr16:87587380-87696813	74	rs16965228	rs4782455	109	Dup	inherited
22009	chr16:87621744-87773456	61	rs11647758	rs2287358	152	Dup	inherited
1095	chr17:271176-439536	82	rs6565724	cnvi0078631	168	Dup	inherited
10048	chr17:1015143-1217312	78	rs7207116	rs7208041	202	Dup	deNovo
19020	chr17:6573151-6739601	39	rs1073031	cnvi0152609	166	Del	inherited
19006	chr17:9761420-10217371	225	rs4791910	rs3809738	456	Dup	inherited
5048	chr17:12732593-12901438	70	rs4791514	rs4792325	169	Dup	inherited

7003	chr17:31559291-31699832	113	cnvi0059395	cnvi0091042	141	Del	deNovo
5025	chr17:31559291-31810869	119	cnvi0059395	cnvi0066212	252	Del	inherited
1038	chr17:31621324-31822399	89	cnvi0081269	cnvi0064051	201	Del	deNovo
4037	chr17:32227936-33270047	408	rs1273149	rs7215724	1042	Dup	inherited
8025	chr17:32788938-32942417	38	rs4795194	rs12603185	153	Del	deNovo
13012	chr17:48301335-48660544	57	rs4374212	rs807081	359	Del	inherited
13013	chr17:48306578-48660544	55	rs4531772	rs807081	354	Del	inherited
4044	chr17:63353899-63494186	30	rs8065275	rs8079936	140	Del	deNovo
21023	chr17:63420046-63720352	57	cnvi0104495	rs12949854	300	Del	inherited
10028	chr17:69350709-70153035	352	rs2620049	rs477963	802	Dup	inherited
27401	chr17:74998649-75290039	66	cnvi0131085	cnvi0114048	291	Dup	deNovo
23010	chr17:75003043-75306582	62	cnvi0120403	cnvi0139891	304	Dup	inherited
22048	chr17:75436486-75693304	101	rs1696745	rs1800300	257	Dup	inherited
5024	chr17:77254717-77521027	48	rs7502521	cnvi0140450	266	Dup	deNovo
5010	chr17:77413243-77569714	30	rs35138785	rs4969472	156	Dup	deNovo
1138	chr17:77413243-77616564	51	rs35138785	rs28631946	203	Dup	inherited
1057	chr17:77517008-77628185	39	rs4239275	rs12949488	111	Dup	deNovo
5053	chr17:77869119-78057994	66	rs10852795	rs9911520	189	Dup	inherited
1098	chr18:7675307-7926616	65	rs660633	rs7232467	251	Dup	inherited
1098	chr18:9224774-9353215	32	rs9960129	rs9957701	128	Dup	inherited
11043	chr18:10587874-11910658	593	rs8089027	rs9962216	1323	Dup	deNovo
5069	chr18:12852942-12976000	35	rs670671	rs7233600	123	Del	deNovo
1140	chr18:23130927-23355550	57	rs7243573	rs10502494	225	Dup	inherited
11007	chr18:25300409-25453287	30	rs9954312	rs2201215	153	Del	inherited
13049	chr18:40201401-40331903	36	cnvi0148539	rs17778071	131	Del	inherited
1087	chr18:59984700-60123704	34	rs17724685	rs17727176	139	Dup	inherited
13060	chr18:63439310-63769578	150	rs875921	rs1551736	330	Dup	inherited
5024	chr19:2172792-2279281	47	rs12462556	rs11666344	106	Dup	deNovo
15005	chr19:3924600-4053449	46	cnvi0137718	rs17851657	129	Dup	inherited
8019	chr19:3924600-4053449	46	cnvi0137718	rs17851657	129	Dup	inherited
11032	chr19:3924600-4025697	42	cnvi0137718	rs11880023	101	Dup	inherited
16010	chr19:6929013-7041222	31	rs252569	rs17775938	112	Del	inherited
1002	chr19:6940127-7054542	33	cnvi0099036	rs2914559	114	Del	inherited
5071	chr19:22083611-22344442	46	rs7260331	rs16999064	261	Del	inherited
18026	chr19:56995958-57309110	122	rs10421380	rs10418739	313	Dup	inherited
15006	chr19:57068764-57309110	81	rs8107969	rs10418739	240	Dup	inherited
1051	chr19:57122551-57309110	61	rs12461478	rs10418739	187	Dup	inherited
22044	chr20:4173573-4597276	193	rs6052456	rs6139516	424	Dup	inherited
21016	chr20:13291679-13552548	48	rs6042051	cnvi0137029	261	Dup	inherited
1048	chr20:30561538-30687676	32	rs293566	rs7268275	126	Dup	inherited
1114	chr20:56208537-56465163	81	rs563431	rs5007291	257	Dup	deNovo

4048	chr20:60072851-60190884	50	cnvi0157507	rs1056885	118	Dup	deNovo
8015	chr20:61618340-61727992	83	rs6010952	rs6089941	110	Dup	deNovo
13049	chr20:61796554-61951093	89	rs3208008	rs4809238	155	Dup	inherited
7023	chr20:61796554-61939170	87	rs3208008	rs6010675	143	Dup	inherited
13050	chr20:61810559-61951093	76	rs1291212	rs4809238	141	Dup	inherited
22048	chr21:14516366-14821591	155	rs389493	rs2064022	305	Dup	inherited
22048	chr21:16285114-16629593	211	rs2205453	rs151579	344	Dup	inherited
10025	chr21:19726751-20132693	90	rs1977966	rs2825860	406	Del	inherited
16003	chr21:22563501-22845294	34	rs2827319	rs2827563	282	Del	inherited
13032	chr21:34625589-34783587	46	rs2834451	rs2834497	158	Del	inherited
8049	chr21:36406812-36514611	50	rs2835278	rs2835321	108	Dup	inherited
13017	chr21:36406932-36514611	49	rs4817775	rs2835321	108	Dup	inherited
11041	chr21:43958296-44081136	78	rs4819296	rs13046826	123	Dup	inherited
15017	chr22:17404651-19795050	1329	rs2073776	rs2845422	2390	Del	deNovo
10023	chr22:17425784-19349273	1101	rs5993487	rs474117	1923	Del	deNovo
25034	chr22:23621076-23813921	74	rs5760712	rs5996834	193	Dup	inherited
22026	chr22:32194817-32314329	31	rs2413187	rs16992592	120	Del	inherited
23010	chr22:35593792-35697482	49	rs729749	rs17749540	104	Dup	deNovo
1124	chr22:48693936-48827020	59	rs7511620	rs137905	133	Dup	deNovo

Table D. 65 medically relevant and CNV-affected genes according to summary by Zarrei *et* al. [7], which were found here in SA offspring CNVs.

Gene symbol	Approved name	CNV type	Phenotype(s)*
ABCC1	ATP binding cassette subfamily C member 1	del/ dup	LD, multiple congenital anomaly, epilepsy, Autism, Schizophrenia
ABCC6	ATP binding cassette subfamily C member 6	del	Pseudoxanthoma elasticum, forme fruste
ACACA	acetyl-CoA carboxylase alpha	del/ dup	LD, behavioral abnormalities, seizures
ALDOA	aldolase, fructose-bisphosphate A	del/ dup	Autism, LD, Developmental delay, speech delay, behavioral problem, …
AMH	anti-Mullerian hormone	del	Persistent Mullerian duct syndrome (PMDS) / Sex ambiguity
BHLHA9	basic helix-loop-helix family member a9	del	Split-hand/foot malformation
CCL3L1	C-C motif chemokine ligand 3 like 1	High/ low CN	HIV/AIDS susceptibility, systemic lupus erythematosus, rheumatoid arthritis, and type 1 diabetes
CCL4L1	C-C motif chemokine ligand 4 like 1		
CCL4L2	C-C motif chemokine ligand 4 like 2		
CDR2	cerebellar degeneration related protein 2	del/ dup	LD, developmental or behavioural problems
CHL1	cell adhesion molecule L1 like		ASD candidate gene
CIT	citron rho-interacting serine/threonine kinase	del	Schizophrenia
CLIP2	CAP-Gly domain containing linker protein 2	del/ dup	Williams-Beuren syndrome
CNTN4	contactin 4	del/ dup	ASD
COMT	catechol-O-methyltransferase	del/ dup	LD, behavioral features, Schizophrenia
CYFIP1	cytoplasmic FMR1 interacting protein 1	del	Idiopathic generalized, Schizophrenia, LD, Behavioral problems, developmental delay, ASD, craniofacial features
DGCR14	DiGeorge syndrome critical region gene 14	del	Mental retardation
DGCR2	DiGeorge syndrome critical region gene 2	del	Schizophrenia
DGCR8	DGCR8 microprocessor complex subunit	del	Schizophrenia
DISC1	disrupted in schizophrenia 1	del	Schizophrenia
DOC2A	double C2 domain alpha		Schizophrenia
DOPEY2	dopey family member 2		Alzheimer's disease
EEF2K	eukaryotic elongation factor 2 kinase	del/ dup	LD, multiple congenital anomaly
ELN	elastin	del/ dup	Williams-Beuren syndrome

EVX2	even-skipped homeobox 2	del	2q31.1 deletion syndrome
FZD9	frizzled class receptor 9	del	Williams-Beuren syndrome
GNB1L	G protein subunit beta 1 like	del/ dup	Schizophrenia, DiGeorge/Velocardiofacial syndrome
GTF2I	general transcription factor IIi	del/ dup	Williams-Beuren syndrome
GTF2IRD1	GTF2I repeat domain containing 1	del	Williams-Beuren syndrome
HNF1B	HNF1 homeobox B	del/ dup	LD, behavioral abnormalities, seizures, mental retardation
HOXD13	homeobox D13	del	Synpolydactyly, HOXD deletion syndrome
IGF1R	insulin like growth factor 1 receptor	del	Psychiatric phenotypes, developmental delay, …
IRGM	immunity-related GTPase M	del/ dup	Crohn's disease
KCNE1	potassium voltage-gated channel subfamily E regulatory subunit 1	dup	Down syndrome critical region
KCNE2	potassium voltage-gated channel subfamily E regulatory subunit 2		
LARGE	LARGE xylosyl- and glucuronyltransferase 1	del	Schizophrenia
LFNG	LFNG O-fucosylpeptide 3-beta-N-acetylglucosaminyltransferase	dup	Asperger Syndrome
LHX1	LIM homeobox 1	del/ dup	LD, seizures
LIMK1	LIM domain kinase 1	del/ dup	Williams-Beuren syndrome
LINGO2	leucine rich repeat and Ig domain containing 2		ASD
MAP2K2	mitogen-activated protein kinase kinase 2	del	Cardio-facio-cutaneous syndrome
MTAP	methylthioadenosine phosphorylase	del	ISCA
MYH11	myosin, heavy chain 11, smooth muscle	del/ dup	Schizophrenia, Autism, LD, …
NCF1	neutrophil cytosolic factor 1	del	Williams-Beuren syndrome
NDE1	nudE neurodevelopment protein 1	del/ dup	Schizophrenia, Autism, LD, …
NIPA1	non imprinted in Prader-Willi/Angelman syndrome 1	del/ dup	Idiopathic generalized, Schizophrenia, LD, Behavioral problems, developmental delay, ASD, craniofacial features
NIPA2	non imprinted in Prader-Willi/Angelman syndrome 2		
NRXN1	neurexin 1	del/ dup	Autism, ADHD, ASD, Schizophrenia, Tourette syndrome
PI4KA	phosphatidylinositol 4-kinase alpha	del	Schizophrenia
POLR3E	polymerase (RNA) III subunit E	del	LD, multiple congenital anomaly
PRRT2	proline rich transmembrane protein 2		ISCA

QPRT	quinolinate phosphoribosyltransferase	del/ dup	Autism, LD, Developmental delay, speech delay, behavioral problems, …
RB1	RB transcriptional corepressor 1		ISCA
RFC2	replication factor C subunit 2	del	Williams-Beuren syndrome
ROR2	receptor tyrosine kinase like orphan receptor 2	del	Robinow syndrome/brachydactyly type B1
SATB2	SATB homeobox 2	del	2q33.1 microdeletion syndrome
SEZ6L2	seizure related 6 homolog like 2	del/ dup	Autism, LD, Developmental delay, speech delay, behavioral problems, …
SLC25A22	solute carrier family 25 member 22	del	Myoclonic epilepsy, neonatal, with suppression-burst pattern
SLC4A10	solute carrier family 4 member 10		ISCA
SRD5A2	steroid 5 alpha-reductase 2		ISCA
STX1A	syntaxin 1A	del	Williams-Beuren syndrome
TBX1	T-box 1	del/ dup	LD, Schizophrenia, Velocardiofacial syndrome, behavioral features, …
TBX6	T-box 6	del/ dup	Autism, LD, Developmental delay, speech delay, behavioral problems, …
YWHAE	tyrosine 3-monooxygenase/tryptophan 5-monooxygenase activation protein epsilon	dup	Microduplication 17p13.3
ZDHHC8	zinc finger DHHC-type containing 8	del	Schizophrenia

CN, copy number; LD, Learning disability; ASD, autism spectrum disorders; ISCA, International Standards for Cytogenomic Arrays (https://www.clinicalgenome.org/) dosage sensitive gene.
* see summary by Zarrei *et* al. [7] for further details and references.

Table E. Significant enrichment of Gene Ontology Biological Process (GO-BP) sets observed using ToppGene, among the 65 medically relevant and CNV-affected genes found in SA offspring CNVs.

ID	Name	pValue	Bonferroni	Genes from Input*	Genes in Annotation
GO:0048667	cell morphogenesis involved in neuron differentiation	6.95E-08	1.32E-04	13	590
GO:0060322	head development	3.53E-07	6.73E-04	14	802
GO:0048699	generation of neurons	3.84E-07	7.31E-04	19	1528
GO:0048806	genitalia development	7.10E-07	1.35E-03	5	50
GO:0022008	neurogenesis	1.01E-06	1.93E-03	19	1628
GO:0048812	neuron projection morphogenesis	1.11E-06	2.12E-03	12	630
GO:0031175	neuron projection development	2.74E-06	5.22E-03	14	953
GO:0048666	neuron development	3.31E-06	6.30E-03	15	1114
GO:0000904	cell morphogenesis involved in differentiation	3.76E-06	7.17E-03	13	840
GO:0007409	axonogenesis	4.04E-06	7.69E-03	10	475
GO:0007420	brain development	7.19E-06	1.37E-02	12	755
GO:0061564	axon development	8.08E-06	1.54E-02	10	514
GO:2000026	regulation of multicellular organismal development	9.46E-06	1.80E-02	19	1893
GO:0009952	anterior/posterior pattern specification	1.04E-05	1.97E-02	7	224
GO:0030182	neuron differentiation	1.16E-05	2.22E-02	16	1398
GO:0001764	neuron migration	1.66E-05	3.16E-02	6	160
GO:0022603	regulation of anatomical structure morphogenesis	1.89E-05	3.59E-02	14	1128
GO:0007417	central nervous system development	2.49E-05	4.74E-02	13	1002

* 29 out of the 65 genes were found among these GO-BPs: *ALDOA, AMH, CHL1, CIT, CNTN4, CYFIP1, DISC1, EEF2K, FZD9, GTF2I, HNF1B, HOXD13, IGF1R, LFNG, LHX1, LIMK1, LINGO2, MAP2K2, NDE1, NRXN1, RB1, ROR2, SATB2, SEZ6L2, SLC4A10, SRD5A2, TBX1, TBX6* and *YWHAE*.

Table F. Genes found within CNV calls in SA offspring here and which contributed to the SNP-based PRS (increased common risk allele burden) in SA and/or in the PRS-overlap with Schizophrenia, in our previous GWAS [9].

Gene symbol	Approved name	PRS neural dev association in SA?	PRS overlap between Schizophrenia and SA?
ADAMTS2	cadherin 15	x	x
CDH15	citron rho-interacting serine/threonine kinase		x
CIT	cell adhesion molecule L1 like		x
CHL1	contactin 4	x	
CNTN4	contactin 6	x	x
CNTN6	dachshund family transcription factor 1	x	x
DACH1	eukaryotic translation initiation factor 2B subunit delta	x	
EIF2B4	EPH receptor A5		x
EPHA5	even-skipped homeobox 2	x	x
EVX2	GLIS family zinc finger 3	x	x
GLIS3	hephaestin like 1	x	x
HEPHL1	homeobox D12	x	
HOXD12	homeobox D13		x
HOXD13	insulin like growth factor 1 receptor		x
IGF1R	interleukin 5 receptor subunit alpha	x	
IL5RA	integral membrane protein 2B	x	
ITM2B	LIM homeobox 1	x	
LHX1	leucine rich repeat and Ig domain containing 2	x	
LINGO2	limbic system-associated membrane protein	x	x
LSAMP	latent transforming growth factor beta binding protein 1	x	x
LTBP1	mastermind like transcriptional coactivator 1	x	
MAML1	mitogen-activated protein kinase 3		x
MAPK3	matrix metallopeptidase 16		x
MMP16	neurexin 1		x
NRXN1	protein phosphatase, Mg2+/Mn2+ dependent 1G	x	x
PPM1G	protein tyrosine phosphatase, receptor type M		x
PTPRM	RAN binding protein 1		x
RANBP1	receptor tyrosine kinase like orphan receptor 2		x
ROR2	RAR related orphan receptor A		x
SDK1	sidekick cell adhesion molecule 1		x
SGSM1	small G protein signaling modulator 1		x
TBC1D9B	TBC1 domain family member 9B		x
TNR	tenascin R		x

Supplementary references

1. Chapman J, Rees E, Harold D, Ivanov D, Gerrish A, Sims R et al. A genome-wide study shows a limited contribution of rare copy number variants to Alzheimer's disease risk. Human molecular genetics. 2013;22(4):816-24.
2. Grozeva D, Conrad DF, Barnes CP, Hurles M, Owen MJ, O'Donovan MC et al. Independent estimation of the frequency of rare CNVs in the UK population confirms their role in schizophrenia. Schizophr Res. 2012;135(1-3):1-7.
3. Rees E, Walters JT, Georgieva L, Isles AR, Chambert KD, Richards AL et al. Analysis of copy number variations at 15 schizophrenia-associated loci. The British journal of psychiatry : the journal of mental science. 2014;204(2):108-14.
4. Stankiewicz TR, Linseman DA. Rho family GTPases: key players in neuronal development, neuronal survival, and neurodegeneration. Frontiers in cellular neuroscience. 2014;8:314.
5. Abramson JH. WINPEPI updated: computer programs for epidemiologists, and their teaching potential. Epidemiologic perspectives & innovations : EP+I. 2011;8(1):1.
6. Szklarczyk D, Franceschini A, Wyder S, Forslund K, Heller D, Huerta-Cepas J et al. STRING v10: protein-protein interaction networks, integrated over the tree of life. Nucleic Acids Res. 2015;43(Database issue):D447-52.
7. Zarrei M, MacDonald JR, Merico D, Scherer SW. A copy number variation map of the human genome. Nat Rev Genet. 2015;16(3):172-83.
8. MacDonald JR, Ziman R, Yuen RK, Feuk L, Scherer SW. The Database of Genomic Variants: a curated collection of structural variation in the human genome. Nucleic Acids Res. 2014;42(Database issue):D986-92.
9. Sokolowski M, Wasserman J, Wasserman D. Polygenic associations of neurodevelopmental genes in suicide attempt. Mol Psychiatry. 2015.
